# Antibodies targeting phage fiber and nozzle proteins impair *Acinetobacter baumannii* phage therapy by blocking infection and promoting immune clearance

**DOI:** 10.1371/journal.pbio.3004009

**Published:** 2026-09-22

**Authors:** Heng Xue, Xinfeng Li, Guibo Rao, Fen Hu, Mingyue Zhong, Paulina Miernikiewicz, Rui Qiu, Sheng Cao, Krystyna Dąbrowska, Hang Yang

**Affiliations:** 1 State Key Laboratory of Virology and Biosafety, Wuhan Institute of Virology, Chinese Academy of Sciences, Wuhan, China; 2 University of Chinese Academy of Sciences, Beijing, China; 3 Key Laboratory of Ministry of Education for Gastrointestinal Cancer, School of Basic Medical Sciences, Fujian Medical University, Fuzhou, China; 4 Hirszfeld Institute of Immunology and Experimental Therapy, Polish Academy of Sciences, Wrocław, Poland; 5 Faculty of Medicine, Wroclaw University of Science and Technology, Wrocław, Poland; 6 Hubei Jiangxia Laboratory, Wuhan, China; Massachusetts Institute of Technology, Howard Hughes Medical Institute, UNITED STATES OF AMERICA

## Abstract

Phage therapy represents a promising strategy to tackle the growing threat of antimicrobial resistance. Increasing evidence has demonstrated that phage-specific antibodies may compromise the efficacy of phage therapy. Nevertheless, little is known about how phage protein specificity of antibodies modulates phage therapeutic outcomes. Herein, we utilized AbP20, a podovirus against *Acinetobacter baumannii*, to explore the effects of antibodies elicited by each structural protein on phage therapy efficacy in a mouse infection model. Intraperitoneal administration of AbP20 once a day for 7 consecutive days induced robust phage-specific antibody responses that impaired phage therapy. Genome-guided antigen screening identified that antibodies elicited by the nozzle and fiber proteins of AbP20, rather than those targeting the portal, capsid, or adaptor proteins, are the dominant drivers of phage therapy failure. Specifically, fiber-specific and nozzle-specific antibodies block bacterial adsorption and genomic injection of AbP20, respectively. Meanwhile, both antibody subsets efficiently induce large phage aggregates and potentiate macrophage phagocytosis via an Fc receptor-independent pathway. An evolved AbP20 variant with improved neutralization escape capacity exhibited comparable antibody-accelerated phagocytosis and host immune clearance, yet partially rescued therapeutic failure caused by neutralizing antibodies. Collectively, this study elucidates that nozzle- and fiber-elicited neutralizing antibodies impair phage therapy via dual synergistic mechanisms, which offers novel insights into the multifaceted modulation of phage-specific antibodies against phage therapeutic efficacy.

## Abstract 2

Phage therapy can fail when the immune system makes antibodies against therapeutic viruses. This study shows that fiber- and nozzle-specific antibodies block phage infection and promote aggregation-driven, Fc receptor-independent phagocytosis.

## Introduction

The increasing emergence of antimicrobial resistance poses a growing global threat to human and veterinary health [1]. Phage therapy employs viruses that target bacteria to treat bacterial infections, representing a highly promising therapeutic strategy in the era of precision medicine. Multiple clinical studies and retrospective cohort analyses have corroborated the safety of phage therapy [2–9]. Recent studies also validated the efficacy of personalized phage therapy in various scenarios, including respiratory tract infections [10], urinary tract infections [11], osteomyelitis [12], and skin and soft tissue infections [13,14]. Although phage therapy exhibits clinical benefits in multiple indications of infection, several challenges have been identified. A major concern lies in the potential impact of the host immune system on therapeutic efficacy [15–17]. While synergistic crosstalk between host immunity and phages can partially facilitate treatment resolution [18], antagonistic immune–phage interactions raise greater clinical concerns, especially following repeated or long-term administration [19]. Numerous studies have demonstrated that phage therapy can trigger the production of protein-specific antibodies, which may compromise its therapeutic efficacy—a phenomenon largely attributed to the development of neutralizing antibodies [20–23]. Of note, a retrospective analysis of phage therapy in humans showed that 38.5% (5/13) of patients developed phage-neutralizing antibodies between 6 and 35 days after phage therapy, yet clinical improvement and bacterial eradication were still achieved in 80% of patients [18]. Occasionally, cross-neutralizing antibodies targeting prophages induced from the host bacterial genome can also precipitate phage therapy failure [24].

Phages are complex particles composed of numerous proteins and can induce specific antibodies against various phage components regardless of the administration route—whether intraperitoneal, intramuscular, intravenous, or oral [25]. However, it is still difficult to predict the effect that particular protein-specific antibodies may exert on phage activity in vivo. In one study, the titer of antibodies specific to the head of a PB1-like *Pseudomonas* phage was found to be intermediate between those of neutralizing-negative and -positive sera [26], suggesting that head-specific antibodies may not be involved in phage neutralization. Similarly, in another study where mice were immunized with the head protein, tail protein, and baseplate of the staphylococcal phage A3R, phage-neutralizing activity was observed only in the serum from baseplate-immunized mice [22]. In contrast, the production of specific antibodies against the head proteins gp23 and Hoc of T4 phage has been shown to elicit effective neutralizing activity [27].

Importantly, antibodies do not exert effects on phages independently; their functional outcomes instead rely on crosstalk with other immune compartments. Following entry into a mammalian host, phage survival and clearance are predominantly governed by the innate immune system. Bacteriophages are readily internalized by professional phagocytes, including macrophages, dendritic cells, and neutrophils [25–27]. Phage-derived nucleic acids or proteins can activate downstream signaling pathways, stimulate proinflammatory cytokine secretion, and initiate local inflammatory reactions; these inflammatory factors can, in turn, influence the function of phagocytes [28,29]. As central cellular constituents of immune-rich organs such as the spleen and liver, phagocytes serve as the primary in vivo "phage sinks" [22,28,29] and constituent a critical checkpoint for priming antigen-specific immunity. Internalized phage antigens undergo proteolytic processing and are displayed on cell surfaces via major histocompatibility complex (MHC) molecules. T cells engage MHC-peptide complexes through T cell receptors (TCRs), undergo full cellular activation, and mount robust antigen-specific adaptive immune responses. Moreover, antigen-opsonized antibodies can be efficiently captured by phagocytes via Fcγ receptors, which potently triggers engulfment of antibody-coated phage particles. This mechanism substantially elevates the specificity and efficiency of phagocytic clearance and is defined as antibody-dependent cellular phagocytosis (ADCP) [30]. Therefore, even phage-specific nonneutralizing antibodies can potentially promote macrophage-mediated phagocytosis via opsonization, leading to rapid phage degradation [30–32]. However, the adaptive immune response against phages is predominantly dictated by their immunogenic structural proteins [27,33–35]. It is also believed that different structural proteins within the same phage may differ in immunogenicity, leading to heterogenicity in antibody levels. Conversely, certain proteins, despite their lower copy numbers, have been shown to possess high immunogenicity sufficient to influence the fate of phages. For example, Hoc, one of the head proteins of phage T4, effectively induces specific antibodies even though its copy number per virion is only one-sixth that of the major capsid protein [27]. However, detailed studies on how antibodies targeting different structural components of phages affect the outcome of phage therapy remain insufficient.

In the present study, we adopted *Acinetobacter baumannii* phage AbP20 as a model to evaluate the effects of structural protein-elicited antibodies on phage therapy efficacy in mouse models of *A. baumannii* infection. We further elucidated how these antibodies, targeting different sites on phage virions, affect key functions involved in cellular immune responses and clearance. Based on these observations, we propose neutralizing antibody-mediated phagocytosis as a pivotal protein-specific immune mechanism that dictates the in vivo fate of therapeutic phage.

## Results

### AbP20-induced antibodies impair phage therapy

Phage AbP20 is a lytic podovirus isolated from hospital wastewater in Wuhan, with *A. baumannii* strain Ab28 serving as the propagation host. It presents typical T7-like morphology consisting of an icosahedral capsid and a short tail (Fig 1A). Both bacterial growth inhibition and dynamic bactericidal assays verified that AbP20 exhibits potent bactericidal activity against Ab28 across different MOI gradients (Figs 1B and S1A). Whole-genome sequencing analysis revealed that AbP20 harbors a 42.023 kb double-stranded DNA genome (GSA accession: CRA031834) [36,37], which encodes a total of 49 predicted open reading frames (S1B Fig).

**Fig 1:**
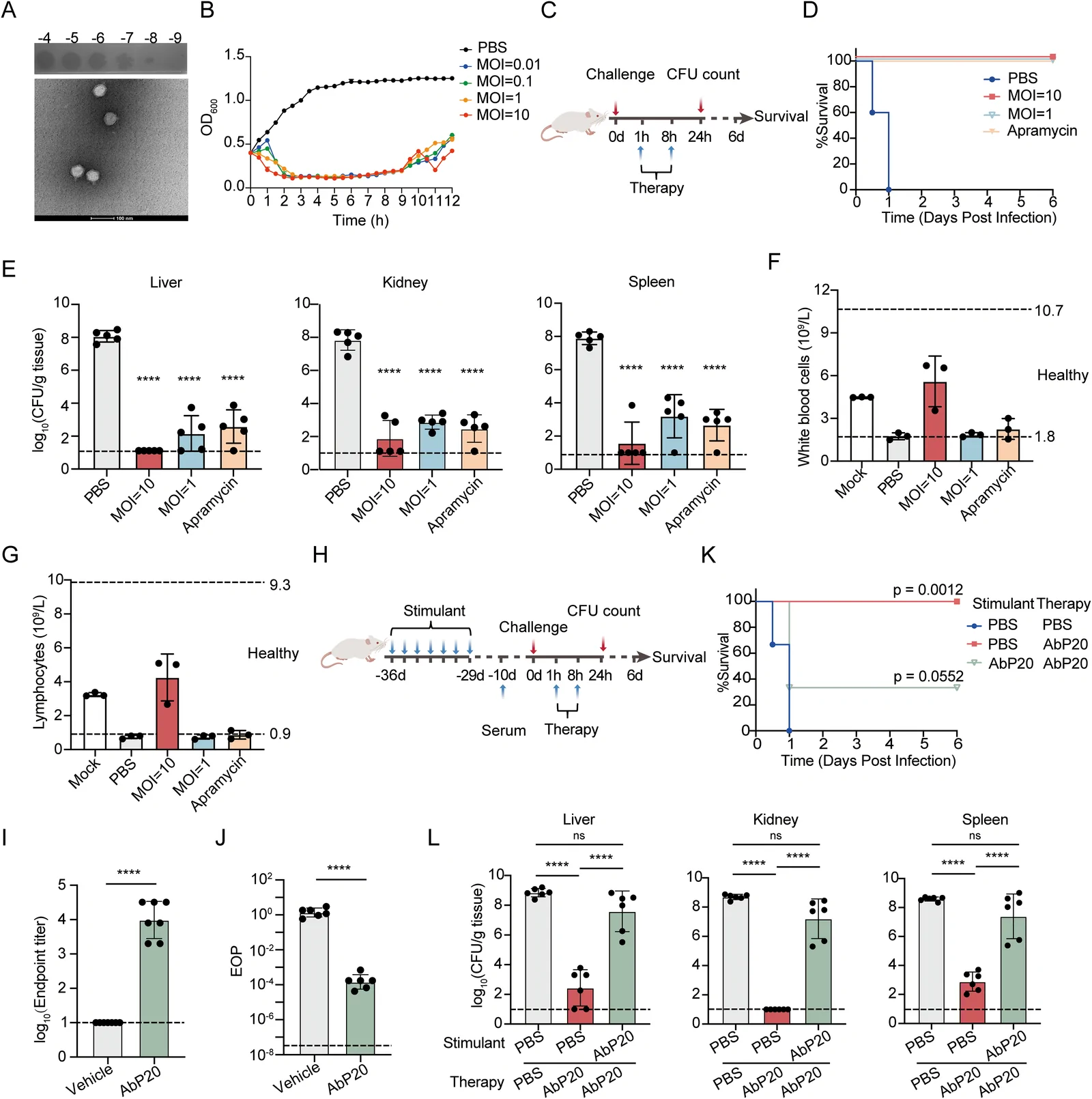
Phage AbP20-induced specific immune responses impair phage therapy. **(A)** Morphology of phage AbP20 and its plaque against *A. baumannii* Ab28. Scalar bar: 100 nm. **(B)** Inhibitory effects of AbP20 on the growth of *A. baumannii* Ab28. Exponential cultures of Ab28 were diluted with LB to an OD600 of 0.4 and incubated with different MOIs (0, 0.01, 0.1, 1, and 10) of AbP20 at 37°C with shaking at 180 rpm. The turbidity of bacterial culture at 600 nm was monitored every 30 min for 12 h. **(C)** Schematic overview of the experimental design in the mouse model of *A. baumannii* Ab28 infection. Mice were infected intraperitoneally with 2 × 10⁸ CFU/mouse of Ab28. Phage AbP20 was administered at 1 h and 8 h post-infection (*n* = 10), survival rare was monitored daily for 6 days. Part of the mice from each group were sacrificed 24 h post-infection for bacterial burden determination (*n* = 5). **(D)** Survival curves in the mouse model of *A. baumannii* infection. Phage AbP20 was administered intraperitoneally with an MOI of 1 or 10. Groups treated with 30 mg/kg apramycin, or an equal volume of PBS served as controls (*n* = 5). **(E)** Bacterial loads in organs from each group collected 24 h post-infection (*n* = 5). **(F-G)** Peripheral blood cell counts of white blood cells (F) and lymphocytes (G) 10 h post-infection in each group (*n* = 3). Blood from mice without *A. baumannii* infection was used as control (Mock). Dashed lines indicate the healthy range. **(H)** Schematic diagram representing a mouse model of *A. baumannii* infection with stimulated phage antibody. Mice were intraperitoneally treated with 100 μL of 1 × 10^10^ PFU/mL AbP20 or an equal volume of PBS daily for 7 consecutive days. Four weeks after the last stimulation, mice were intraperitoneally infected with 2 × 10^8^ CFU/mouse of Ab28. PBS-immunized mice were used as the vehicle control (Vehicle). **(I)** Anti-phage antibody detected by ELISA, with an initial dilution factor of 10. **(J)** Effect of mouse serum on the efficiency of plating (EOP) of phage AbP20. Mouse serum (1:50 diluted) was incubated with 10 μL of 1 × 10^10^ PFU/mL AbP20. The EOP was calculated as the titer of phage incubated with each serum divided by the titer of phage incubated with PBS alone. Dashed line indicates the limit of detection. **(K)** Survival rates in a mouse model of *A. baumannii* infection with pre-exposure to phage AbP20 (*n* = 6). **(L)** Bacterial burden in organs from each group collected 24 h post-infection (*n* = 6). Dashed line indicates the limit of detection. Unpaired Student’s *t* tests. ns: not significant, ****p < 0.0001. The data underlying this figure can be found in S1 Data.

Given the potent bactericidal activity of phage AbP20, we used it as a model to evaluate the effects of phage-specific antibodies on therapeutic outcomes. We first evaluated the therapeutic efficacy of phage AbP20 in a mouse model of Ab28 infection (Fig 1C). To this end, BALB/c mice were intraperitoneally injected with 2 × 10^8^ CFU/mouse of Ab28 and then received an intraperitoneal administration of phage AbP20 at an MOI of 1 or 10 at 1 and 8 h post‑infection. Results showed that all mice in the PBS control group succumbed to death within 24 h post infection (Fig 1D). In contrast, phage-treated animals exhibited a 100% survival rate, comparable to the antibiotic-treated group (*n* = 5, Fig 1D). Specifically, 24 h post-infection, bacterial loads in the liver, spleen, and kidneys were significantly decreased in both the antibiotic-treated group and the phage-treated group (*n* = 5, Fig 1E). Notably, at 10 h post-infection, the high dose AbP20-treated group (MOI = 10, 2 × 10⁹ PFU/mouse) exhibited a balanced hematopoietic cell profile, with proportions of lymphocytes, neutrophils, monocytes, platelets and leukocytes comparable to the uninfected mock group (Figs 1F, 1G and S2), indicating that AbP20 phage at MOI = 10 effectively promotes bacterial clearance and protects mice from lethal *A. baumannii* infection. Moreover, AbP20 exhibited a favorable safety profile after repeated intraperitoneal administration in mice, demonstrating minimal impact on body weight gain, peripheral blood immune cell counts, and organ histopathology (S3 Fig).

Next, we asked whether repeated exposure to AbP20 triggers immune responses that render phage therapy ineffective. To address this issue, mice were administrated 2 × 10⁹ PFU/mouse of AbP20 intraperitoneally once a day for 7 consecutive days and then maintained for another 4 weeks to allow phage-specific antibody generation. Afterwards, mice were infected intraperitoneally with a lethal dose of *A. baumannii* followed by two doses of AbP20 (Fig 1H). Mice were tested for phage-specific antibody levels 10 days before infection. As expected, the serum collected from the phage-stimulated group contained high level phage-specific antibody (Fig 1I), which reduced the efficiency of plating (EOP) of phage AbP20 by four orders of magnitude (Fig 1J), indicating strong neutralization activity. In the subsequent infection experiment, administration of 2 × 10⁹ PFU/mouse of AbP20 only showed 33.3% protection (Fig 1K) and limited bacterial clearance efficacy (Fig 1L) in the phage-stimulated group (n = 6). These results collectively indicate that phage-induced specific immune responses hinder bacterial clearance in vivo.

### Fiber- and nozzle-induced antibodies lead to the failure of phage therapy

Genome-based analysis showed that phage AbP20 contains five structural proteins, i.e., the capsid protein (gp37), the portal protein (gp35), the adaptor protein (gp39), the nozzle protein (gp40), and the fiber protein (gp44) (Fig 2A). To identify which phage protein-elicited antibodies are capable of impairing phage therapy, we expressed genes coding for the five structural proteins using an *E. coli* expression system (S4A Fig). Afterwards, BALB/c mice were intramuscularly immunized with each structural protein to induce sufficient protein-specific antibodies, and the therapeutic efficiency of AbP20 was evaluated in a mouse model of *A. baumannii* infection (Fig 2B). Similar to the AbP20-challenged group, each individual phage protein elicited strong humoral immune responses as determined 3 days before *A. baumannii* infection (S4B Fig) with minor differences among varied IgG types (S4C–S4F Fig). Strong serological cross-reactivity was observed between sera from mice challenged with each structural protein and whole phage particle, indicating that the proteins produced from the expression system were antigenically relevant to native proteins present in the phage virion (Fig 2C). Concordantly, immunization with phage AbP20 also induced antibodies reactive against each structural protein (Fig 2D). Notably, the proteins gp40 and gp44 exhibit high cross-reactivity with the intact phage AbP20, although the capsid protein (gp37) is the most abundant component within the phage virion, suggesting differences in immunogenicity among phage structural components.

**Fig 2:**
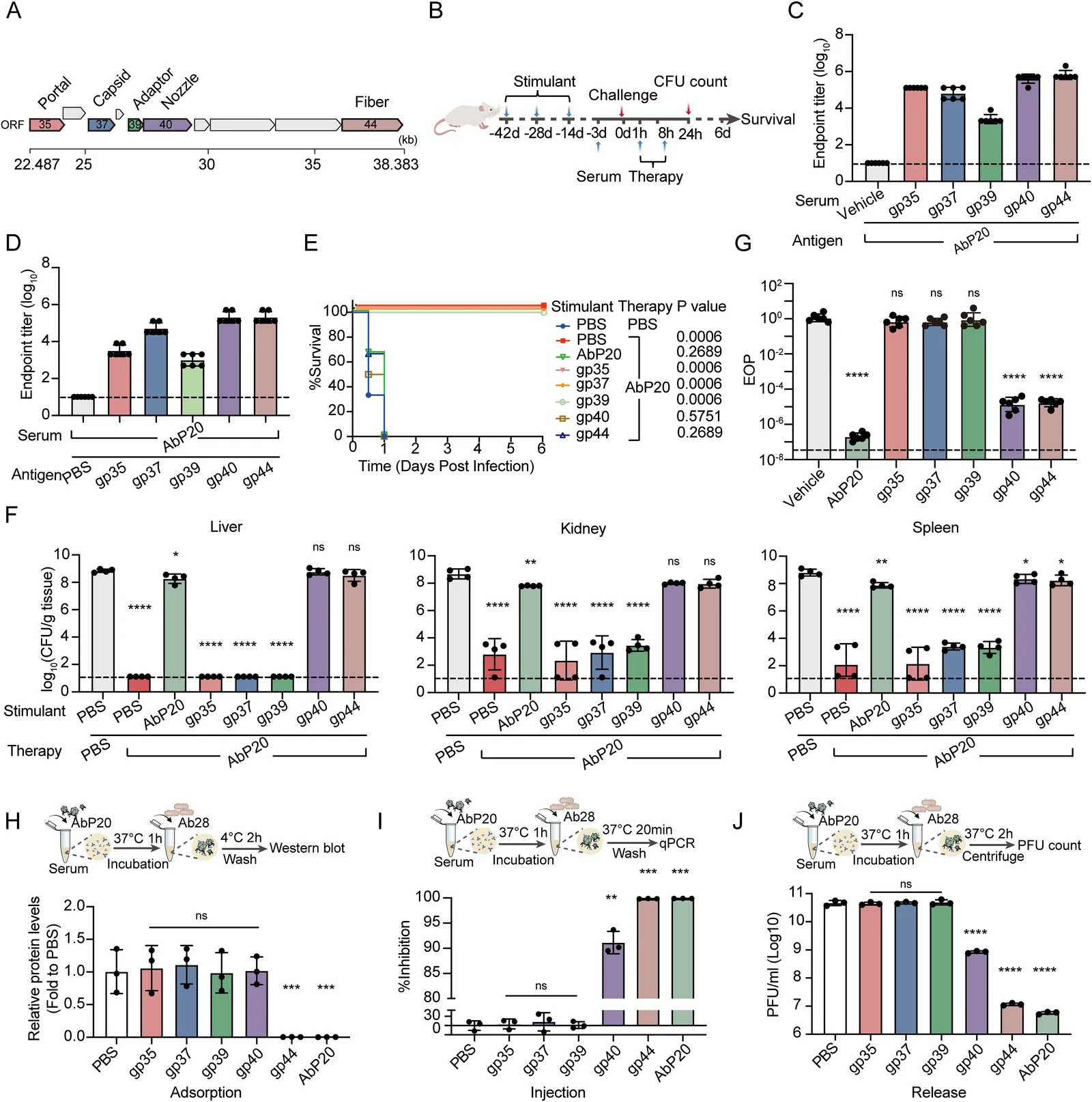
Fiber- and nozzle-induced antibodies lead to the failure of phage therapy. **(A)** Schematic diagram of the structural proteins of phage AbP20. Position of the open reading frames (ORFs) encoding the portal protein gp35, the capsid protein gp37, the adaptor protein gp39, the nozzle protein gp40, and the fiber protein gp44 are shown in different colors in the genome of phage AbP20. **(B)** Schematic diagram of the mouse model investigating the impact of phage structural components on therapeutic efficacy. Mice received three intramuscular immunizations of 10 μg/mouse of either single phage structural protein or 5 × 10^10^ PFU/mouse of phage AbP20 at 14-day intervals. Serum was collected from each group 3 days prior to bacterial challenge for antibody titer detection. Serum from the PBS-stimulated mice were used as the vehicle control (Vehicle). **(C)** IgG titers against AbP20 (Antigen) in varied immune serum (Serum). **(D)** IgG titers against individual structural protein (Antigen) in AbP20-immunized serum (Serum). **(E)** Survival rates of phage therapy (*n* = 6). The immunization strategy, treatments, and *P*-values in each treatment compared to the PBS-treated group are indicated. **(F)** Bacterial burden in liver, spleen, and kidney 24 h post-infection (*n* = 4). **(G)** Effects of varied immune serum on the efficiency of plating (EOP) of phage AbP20. **(H)** Effect of serum against different components of AbP20 on phage adsorption. Serum diluted 100‑fold were incubated with 10⁸ PFU of AbP20 at 37 °C for 1 h, followed by incubation with 10⁸ CFU/mL of Ab28 at 4 °C for 2 h. After washing with PBS, the samples were analyzed by Western blotting. Gray values were calculated using ImageJ software and statistically analyzed by comparing to the PBS-treated group. **(I)** Effect of serum against different components of AbP20 on phage DNA injection. Sera diluted 100‑fold were incubated with 10⁸ PFU of AbP20 at 37 °C for 1 h, followed by incubation with 10⁸ CFU/mL of Ab28 at 37 °C for 20 min. After washing with PBS, the copies were determined by qPCR, and the inhibition rate was calculated and statistically analyzed by comparing to the PBS-treated group. **(J)** Effect of serum against different components of AbP20 on phage release. Serum diluted 100‑fold was incubated with 10⁸ PFU of AbP20 at 37 °C for 1 h, followed by incubation with 10⁸ CFU/mL of Ab28 at 37 °C for 2 h. After centrifugation, phage titers in the supernatant were determined using the double‑layer agar plate method and statistically analyzed by comparing to the PBS-treated group. Dashed line indicates the limit of detection. Unpaired Student’s t tests. ns: not significant, *p < 0.05, **p < 0.01, ***p < 0.001, and ****p < 0.0001.

In the subsequent challenge assay, the same as the phage-stimulated group, immunization with either gp40 or gp44 led to therapeutic failure, whilst immunization with gp37, gp35, and gp39 did not affect the efficacy of phage therapy (*n* = 6, Fig 2E). In line with these observations, phage AbP20 failed to remove *A. baumannii* from organs in groups stimulated with either AbP20, gp40, or gp44 as did in groups stimulated with gp35, gp37, and gp39 (*n* = 4, Fig 2F). Further analysis showed that, although slightly weaker than those induced by the intact phage particles, sera from gp40- and gp44-immunized groups, but not from the gp35-, gp37-, and gp39-immunized groups, exhibited strong phage neutralization capabilities and markedly suppressed phage proliferation (Figs 2G and S5A, S5B). Specifically, fiber protein gp44-elicited antibodies blocked the adsorption of phage AbP20 onto host bacterial cells (Figs 2H and S5C), whereas gp40-specific antibodies selectively inhibited phage genomic injection without interfering with bacterial adsorption (Fig 2I). Such two distinct inhibitory modes ultimately caused a sharp decline in progeny phage virion release (Fig 2J). Altogether, antibodies induced by the fiber and nozzle proteins of AbP20 are probably the major antigenic components of the phage virion that are responsible for inducing antibodies impairing therapeutic outcome.

The data underlying this figure can be found in S1 Data.

### Fiber- and nozzle-induced antibodies promote the clearance of AbP20 by facilitating phagocytosis

It is anticipated that phage-specific antibodies may promote the immune clearance of phages in vivo through multiple mechanisms, one of these being antibody-mediated opsonophagocytosis. Therefore, we used murine macrophage RAW264.7 cells to examine the influence of each structural protein-induced antibody on the phagocytosis of AbP20. In this regard, phages (with a final endotoxin level of 76.23 EU/ml to minimize their effects on macrophage cells) were preincubated with serum from the mice immunized with either a single structural protein or the entire AbP20 at 37 °C for 1 h, after which the mixture was cocultured with macrophage cells for phagocytosis analysis (Fig 3A). Phage engulfment by the cells was assessed by immunodetection of the phage capsid protein (gp37) using rabbit anti-gp37 antibodies. Compared to the PBS-treated group, the mass of gp37 within cells was significantly increased in the presence of AbP20-specific serum up to dilution factor of 320 folds (Fig 3B, 3C), suggesting that AbP20-immunized serum promotes the phagocytosis of AbP20 by macrophages. This conclusion was further supported by confocal fluorescence microscopy (S6 Fig). We then examined whether the antibodies induced by individual structural proteins could promote phage uptake. To our surprise, antibodies induced by the nozzle protein (gp40) and the fiber protein (gp44) significantly enhanced the phagocytosis of AbP20 by macrophage (Fig 3D, 3E). In contrast, antibodies induced by other structural proteins, including the portal protein (gp35), gp37, and the adaptor protein (gp39), did not exhibit significant effects on phage phagocytosis (Fig 3D, 3E). Confocal microscopy further illustrated that gp40 and gp44 immune sera markedly enhanced the internalization of AbP20 by macrophage (Fig 3F), with significantly increased intracellular fluorescence intensity (Fig 3G), whilst differences were not observed in groups treated with gp35-, gp37-, and gp39-specific serum as compared to the native serum (Fig 3F, 3G).

**Fig 3:**
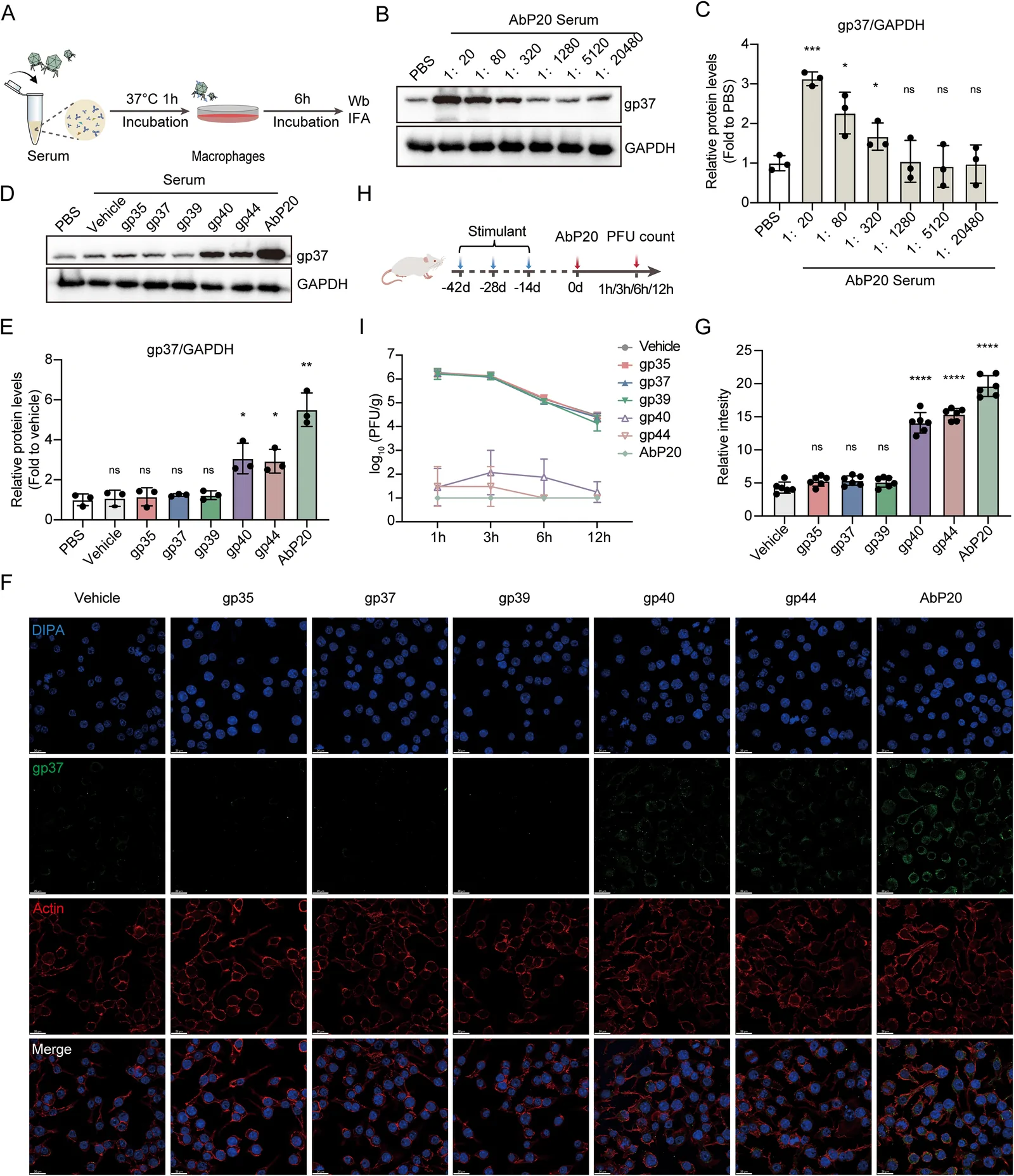
Fiber- and nozzle-specific antibodies promote the phagocytosis of AbP20 by macrophages. **(A)** Schematic overview of the phagocytosis assay. Following incubating phage AbP20 with varied diluted sera for 1 h, the mixture was then moved to incubate with macrophage RAW264.7 cells for 6 h. Phagocytic efficiency was evaluated using Western blotting (Wb) and immunofluorescence assays (IFA). **(B-C)** Effects of AbP20 immune serum on the phagocytosis of AbP20 determined by Wb. Intracellular phage was detected by rabbit anti-gp37 antibodies, using GAPDH as the reference (B). The intensity of gp37 band from three independent Western blots were analyzed by Image J and compared to that of the PBS-treated control (C). **(D-E)** Effects of each structural protein-immune serum (1:20 diluted) on the phagocytosis of AbP20 by macrophages determined by Wb. Serum from PBS-stimulated mice were used as vehicle control (Vehicle). Intracellular phage was detected by rabbit anti-gp37 antibodies, using GAPDH as reference (D). The intensity of gp37 band from three independent Western blots were analyzed by Image J and compared to that of the PBS-treated control (E). **(F-G)** IFA detection of phagocytosis of AbP20 by macrophages. Phage AbP20 is labeled by rabbit anti-gp37 antibodies and CoraLite 488 dye (green), β-actin stained with ActinRed 555 (red), and the nucleus stained with DIPA (blue) (F). The mean fluorescence intensity was quantitative analyzed using LAS X software, with measurements taken from five distinct regions for statistical purposes (G). Scale bar: 20 μm. **(H)** Schematic diagram presenting the workflow for the evaluation of the effects of antibodies on the clearance of phage AbP20 in vivo. Group immunized with PBS served as control (Vehicle). **(I)** Titer of phage AbP20 in spleens from mice immunized with either individual structural protein, entire AbP20, or an equal volume of PBS. All mice received a single dose of AbP20 intraperitoneally 14 days post the last boost. Spleens were collected at 1-, 3-, 6-, and 12-h post-injection for phage titer determination (*n* = 3). Unpaired Student’s *t* tests. ns: not significant, *p < 0.05, **p < 0.01, ***p < 0.001, and ****p < 0.0001. The data underlying this figure can be found in S1 Data. The uncropped blots can be found in S1 Raw Images.

To further validate this assumption, we assessed the clearance of AbP20 in BLAB/c mouse positive to antibodies elicited by each single structural protein, or the entire phage AbP20 (*n* = 3, Fig 3H). Results showed that significantly reduced phage titers were detected in spleens from groups immunized with gp40, gp44, or entire phage AbP20 in all time points detected as compared to groups immunized with gp35, gp37, and gp39 (Fig 3I). Altogether, these findings demonstrate that antibodies elicited by the nozzle and fiber proteins of AbP20 destroy phage activity via dual mechanisms: suppressing phage propagation and potentially promoting immune clearance, thereby impairing phage therapy. By contrast, antibodies elicited by the remaining structural proteins of AbP20 exert negligible impacts on the outcome of phage therapy.

### Antibody‑induced phage agglutination modulates phage phagocytosis

Since the phagocytotic efficiency is governed by the size and shape of cargoes [38], the fact that AbP20-elicited antibodies promote the phagocytosis of AbP20 by macrophage led us to think whether phage-specific antibodies induce the agglutination of phages and thereby affecting their phagocytosis. To investigate this, we first determined the size of phage in the presence of varied immune serum. As expected, enlarged sizes of AbP20 aggregates were observed in the presence of serum immunized with AbP20, the nozzle protein (gp40), and the fiber protein (gp44), but not in the serum immunized with the portal protein (gp35) and the adaptor protein (gp39), as compared to the PBS-treated group (Fig 4A), suggesting that gp40- and gp44-elicited antibodies induced the agglutination of AbP20 and thus facilitating its phagocytosis.

**Fig 4:**
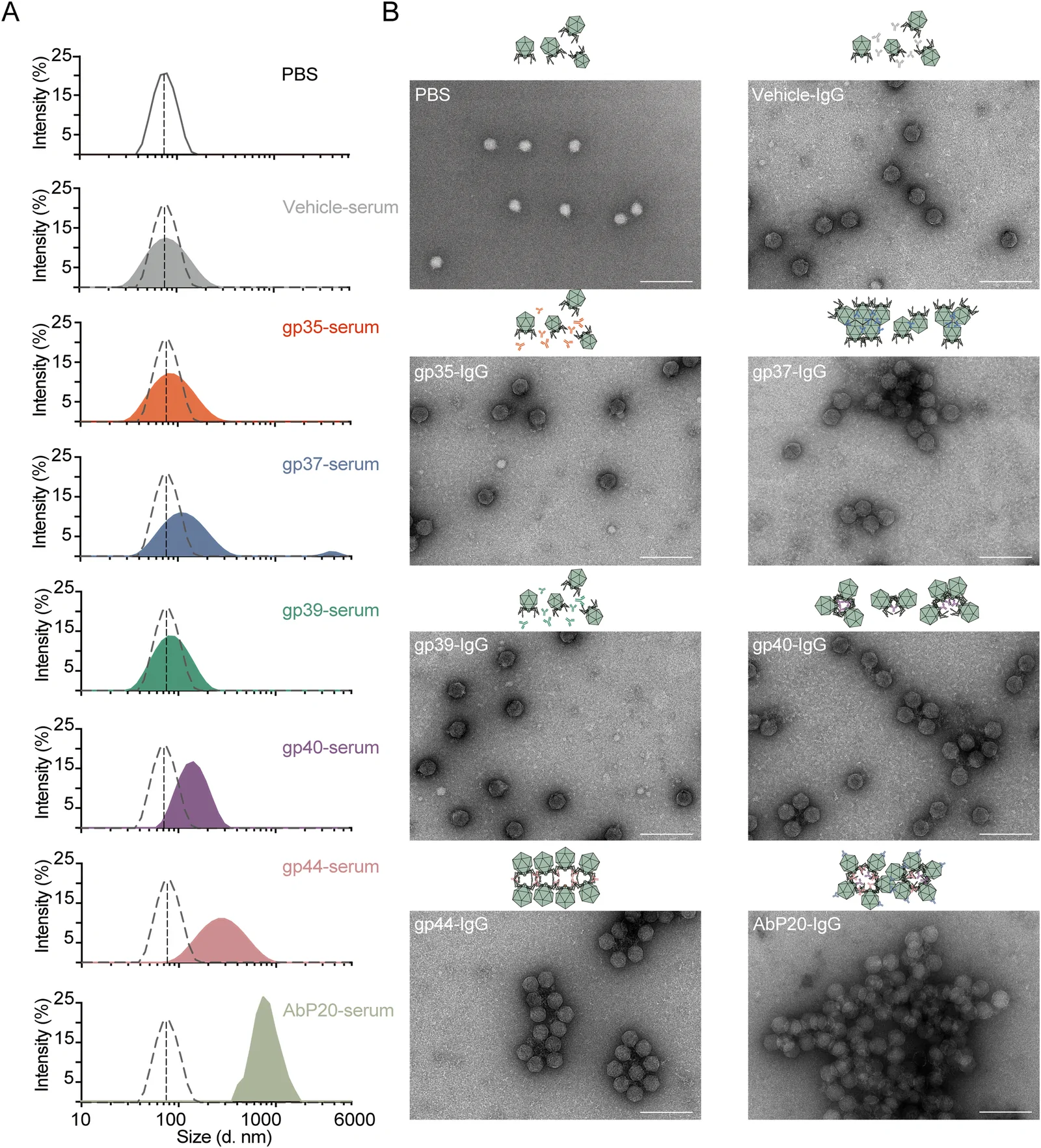
Antibody‑induced phage agglutination modulates phagocytosis. **(A)** Diameter distribution of AbP20 aggregates in the presence of varied immune serum (1:20 diluted). The intensity profile of PBS-treated group was shown in dashed line in the other panels as a reference. **(B)** Representative images of AbP20 aggregates in the presence of varied IgGs purified from varied immune serum. Model manners of phage conjugation under each condition are presented above each image. Scalar bar: 200 nm. IgG from the PBS-immunized mice were used as vehicle control (Vehicle). The data underlying this figure can be found in S1 Data.

To make it clear, we purified IgG with high titers from different immune sera using protein G resin (S7A Fig) and found that, resembling the immune serum, purified IgG against gp40, gp44, and the whole phage particle exhibited strong neutralizing activity and promoted phage phagocytosis by macrophages (S7B, S7C Fig). We then detected the status of AbP20 aggregates in the presence of varied IgG using transmission electron microscopy. As shown in Fig 4B, varied sizes of aggregates were indeed observed in the presence of IgG obtained from AbP20-, gp40-, gp44-, and gp37-immunized serum, whilst aggregates were not observed in the presence of IgG obtained from gp35-, gp39-, and the PBS-immunized serum (Vehicle). Detailed observation of antibody-mediated phage aggregates revealed that the aggregation pattern induced by antibodies targeting gp37 differs from those induced by antibodies targeting gp40 and gp44 (S8 Fig), since in principle, head-targeting antibodies may trigger phage assembly into structures featuring head-to-head juxtaposition, with the spike-like phage tails exposed outward. In contrast, antibodies targeting tail components may induce tail-to-tail phage clustering, where phage spherical capsids oriented toward the exterior.

### Size of antibody-induced phage aggregates regulates phagocytosis in an Fc receptor-independent manner

It is well established that Fc receptor engagement facilitates phagocytosis and immune clearance via multiple effector pathways, including antibody‑dependent cellular phagocytosis [39]. Antibodies elicited by the capsid protein (gp37) exhibited a comparable serotype profile (S4 Fig) but exerted disparate effects on macrophage-mediated phagocytosis (Fig 3E), which prompted us to explore the role of Fc receptor-mediated phagocytosis in this process. To this end, we examined the effects of structural protein-specific immune serum on RAW264.7 cell-mediated phagocytosis of AbP20 upon Fc receptor blockade. Intriguingly, no obvious disparities in antibody-triggered phagocytosis were detected between the Fc receptor-blocked groups (FcR-block) and isotype control groups (Fig 5A), revealing that the nozzle protein (gp40)- and fiber protein (gp44)-specific antibodies accelerate AbP20 phagocytosis in an Fc receptor-independent manner.

**Fig 5:**
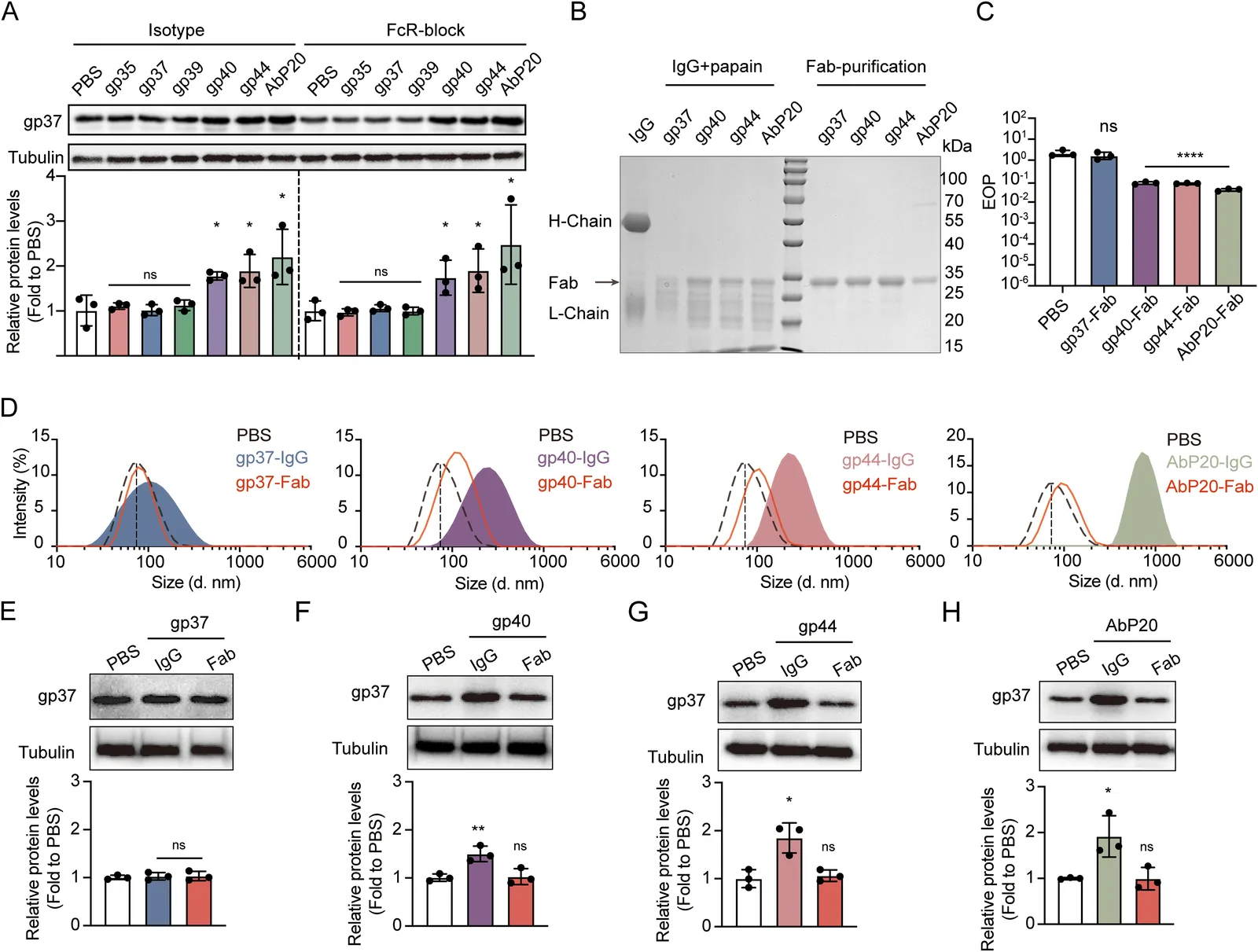
Size of antibody-induced phage agglutination regulates phagocytosis in an Fc receptor-independent manner. **(A)** Effect of immune serum on the phagocytosis of AbP20 by macrophages in the presence of Fc receptor blocker. Intracellular phages after incubation with Fc receptor blocker (Anti‑mouse CD16/CD32) or its isotype were detected using rabbit anti‑gp37 antibody, with Tubulin serving as a reference. Band intensities of gp37 from three independent Western blots were analyzed by Image J software and compared with those of the PBS‑treated control group. **(B)** Preparation of IgG Fab fragments. Papain and purified IgGs were mixed at a ratio of 1:10 and incubated at 37 °C for 12 h. After purification, Fab fragments were obtained and verified by SDS‑PAGE analysis. **(C)** Effects of varied Fabs on the efficiency of plating (EOP) of phage AbP20. **(D)** Diameter distribution of AbP20 aggregates in the presence of different Fabs or IgGs. The intensity distribution of the PBS‑treated group is shown as a dashed line, serving as a reference for the other groups. **(E–H)** Effect of Fabs or IgGs on AbP20 phagocytosis by RAW264.7 cells. Intracellular phages were detected using rabbit anti‑gp37 antibody, with Tubulin serving as a reference. Band intensities of gp37 from three independent Western blots were analyzed by Image J software and compared with those of the PBS‑treated control group. Unpaired Student’s *t* tests. ns: not significant, *p < 0.5, **p < 0.01, and ****p < 0.0001. The data underlying this figure can be found in S1 Data. The uncropped blots and gels can be found in S1 Raw Images.

We next investigated whether antibody-potentiated phagocytosis is solely driven by the formation of large phage aggregates. For this purpose, we purified Fab fragments targeting gp37, gp40, gp44, and intact AbP20 via papain digestion of corresponding purified IgGs (Fig 5B). After verifying the neutralizing activity of all prepared Fab fragments (Fig 5C), we assessed their impacts on phage agglutination and subsequent macrophage phagocytosis. Results showed that phage aggregates formed in the Fab-treated groups were drastically smaller than those formed in the full-length IgG-treated groups (Fig 5D). Consistently, the enhanced RAW264.7 phagocytosis of AbP20 induced by gp40-specific, gp44-specific and AbP20-specific IgGs was abrogated after treatment with their matching Fab fragments (Fig 5E–5H). Taken together, these findings indicate that antibody-mediated formation of large phage aggregates acts as the predominant driver of enhanced macrophage phagocytosis of therapeutic phages, wherein the IgG Fc domain plays an indispensable regulatory role that requires further mechanistic exploration.

### Evolved neutralization escape partially ameliorates antibody-mediated impairment of phage therapy

All the above observations indicate that antibodies elicited by the nozzle protein gp40 and the fiber protein gp44 impair phage therapy via potent neutralization and enhanced phagocytosis of AbP20. As a means to delineate the contribution of each effector mechanism, we conducted in vitro phage evolution by incubating wild-type AbP20 with serially diluted AbP20-immune serum for a total of 10 passages (Fig 6A). Consequently, we obtained a phage variant harboring mutation in the nozzle protein gp40 (Y305H and T656G) and the fiber protein gp44 (Q131K), designated AbP20M (Fig 6B). This mutant exhibited enhanced neutralization escape capability (Fig 6C) and retained comparable bacteriostatic activity relative to wild-type AbP20 (S9A Fig). We further evaluated the responsiveness of AbP20M to antibody-mediated agglutination and macrophage phagocytosis. Results demonstrated that IgGs purified from serum against the capsid protein (gp37-IgG), nozzle protein (gp40-IgG), fiber protein (gp44-IgG), and intact AbP20 (AbP20-IgG) triggered AbP20M aggregation with morphological features and aggregate sizes comparable to those of wild-type AbP20 (Fig 6D, 6E). Consistent with these results, RAW264.7 macrophage cells displayed nearly identical phagocytic efficiency toward free AbP20M and wild-type AbP20 (S9B Fig). Resembling wild-type AbP20, AbP20M phagocytosis by RAW264.7 cells was also markedly promoted by gp40-IgG, gp44-IgG and AbP20-IgG (Fig 6F–6H). Next, we assessed the in vivo clearance rate of AbP20M in mice immunized against each individual structural protein or intact AbP20 (n = 3). Results revealed that the time-dependent splenic clearance of AbP20M in mice immunized with gp40, gp44 or phage AbP20 was markedly lower than that in the other groups (S9C Fig), which was consistent with the clearance pattern of wild-type phage AbP20. These observations collectively suggest that the evolved variant AbP20M confers enhanced neutralization escape capacity while possessing equivalent immune clearance properties relative to wild-type AbP20.

**Fig 6:**
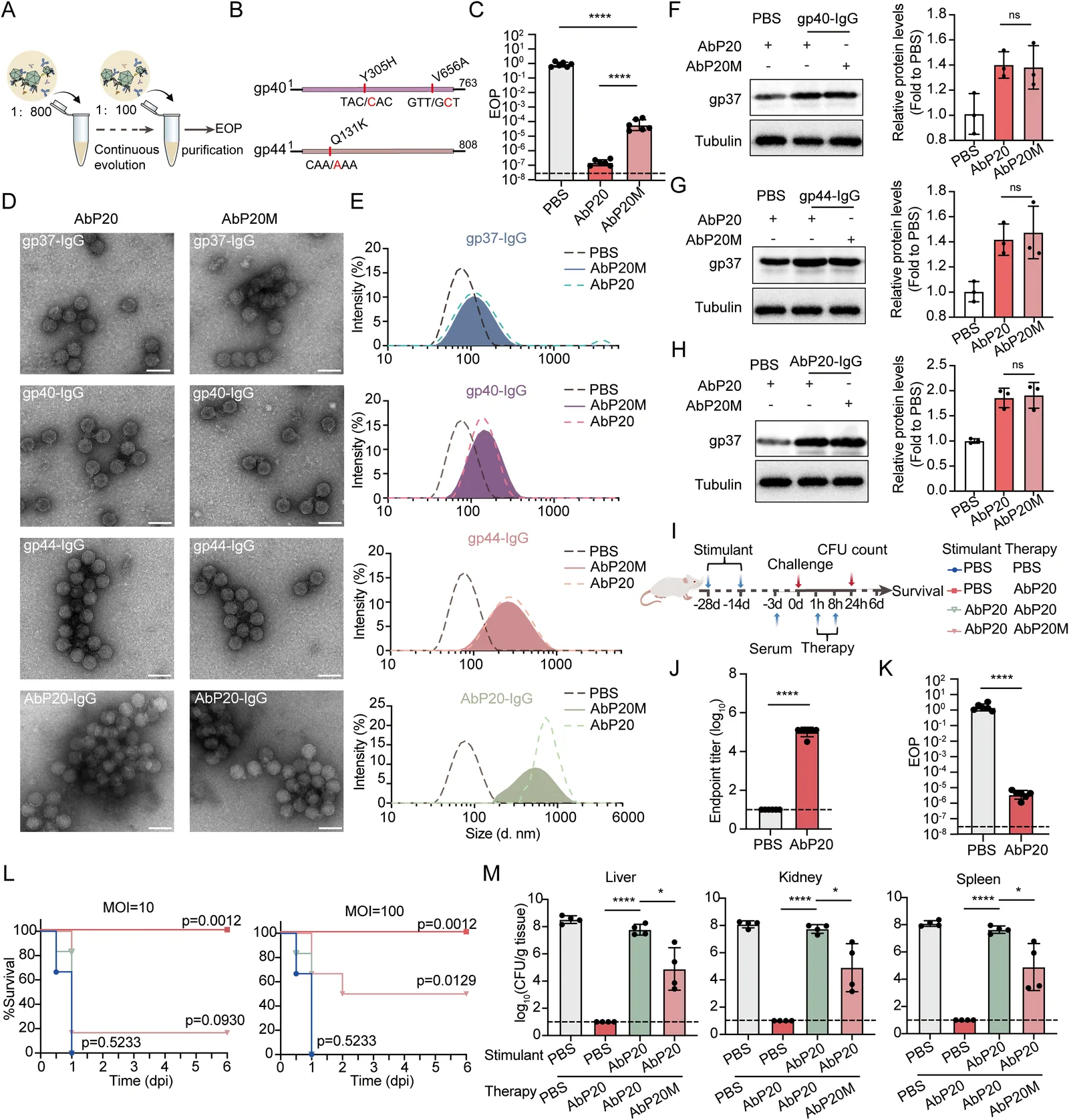
AbP20M with improved neutralization escape ability ameliorates antibody-mediated therapeutic damage. **(A)** Schematic diagram showing the in vitro evolution of AbP20. AbP20‑immunized serum was serially diluted from 1:800 to 1:100 (with triplicate screening at 1:100), incubated with phage AbP20, and allowed to infect *A. baumannii* Ab28; 10 plaques were picked per round for serial passage under increasing selection pressure for EOP and sequencing analysis. **(B)** The mutation sites of AbP20M. **(C)** AbP20M shows significantly enhanced antibody-induced neutralization escape efficiency. **(D)** Representative images of AbP20M and AbP20 aggregates in the presence of varied IgGs. Scalar bar: 100 nm. **(E)** Size distribution of AbP20M aggregates in the presence of varied IgGs. The intensity distributions of the PBS‑treated group and AbP20 aggregates are indicated by dashed lines in different colors. **(F–H)** Effect of varied IgGs on the phagocytosis of AbP20M and AbP20 by RAW264.7 cells. Intracellular phages were detected using rabbit anti‑gp37 antibody, with Tubulin serving as a reference. Band intensities of gp37 from three independent Western blots were analyzed by Image J software and compared. **(I)** Schematic diagram presenting the evaluation of AbP20M in mouse model. Mice received two rounds of intramuscular immunization. Serum was collected 3 days prior to bacterial challenge for antibody titer detection. Subsequent treatment and sampling procedures were consistent with the previous protocol (*n* = 6). **(J)** Anti-phage IgG antibodies detected by ELISA, with an initial dilution factor of 10. **(K)** Neutralization effects of AbP20-immune serum detected by EOP assay. (**L)** Survival rates of mice in a bacteremia model. Mice were immunized with wild‑type AbP20 and subsequent treatment with varied dose of AbP20 or AbP20M. **(M)** Bacterial burden in the liver, spleen, and kidneys from mice receiving 100 MOI AbP20M treatment (*n* = 4). Dashed line indicates the limit of detection. Unpaired Student’s *t* tests. ns: not significant, *p < 0.05, and ****p < 0.0001. The data underlying this figure can be found in S1 Data. The uncropped blots can be found in S1 Raw Images.

Furthermore, we evaluated the therapeutic efficacy of AbP20M in an *A. baumannii*-infected mouse model pre-immunized with intact AbP20 prior to phage therapy (*n* = 6, Fig 6I). As expected, intramuscular immunization elicited high antibody titers against AbP20 (Fig 6J), which exerted potent neutralizing activity (Fig 6K). Accordingly, negligible protection was observed in mice treated with wild-type AbP20 at doses of 10 or 100 MOI (Fig 6L), indicating that AbP20-elicited antibodies thoroughly impair phage therapeutic effects. In contrast, dose-dependent protective effects were observed in AbP20M-treated groups. Specifically, one of six mice survived in the 10 MOI AbP20M treatment group (Fig 6L), while the survival rate rose to 50% at an AbP20M dose of 100 MOI (Fig 6L), accompanied by markedly reduced tissue bacterial burden (Fig 6M). Collectively, these results demonstrate that the evolutionary acquisition of resistance to phage-elicited antibody neutralization can partially improve phage therapy efficacy; nevertheless, antibody-mediated phagocytosis and immune clearance remain major barriers that cannot be fully overcome.

Based on all these observations, we propose a working mechanistic scenario for antibody-mediated phage therapy outcomes. Antibodies targeting distinct structural proteins of phage AbP20 exert disparate regulatory effects on phage fate and therapeutic performance. Specifically, antibodies against the capsid protein (gp37), portal protein (gp35), and adaptor protein (gp39) mainly serve as binding antibodies, which exert negligible impacts on phage infectivity and macrophage-mediated phagocytosis, and barely alter final phage therapeutic efficacy. By comparison, antibodies induced by the fiber protein (gp44) and nozzle protein (gp40) act as dominant neutralizing antibodies. These two types of antibodies suppress phage proliferation via blocking phage-host interaction and accelerate systemic phage clearance by triggering the formation of large phage aggregates, thereby compromise the therapeutic potency of phage AbP20 (Fig 7).

**Fig 7:**
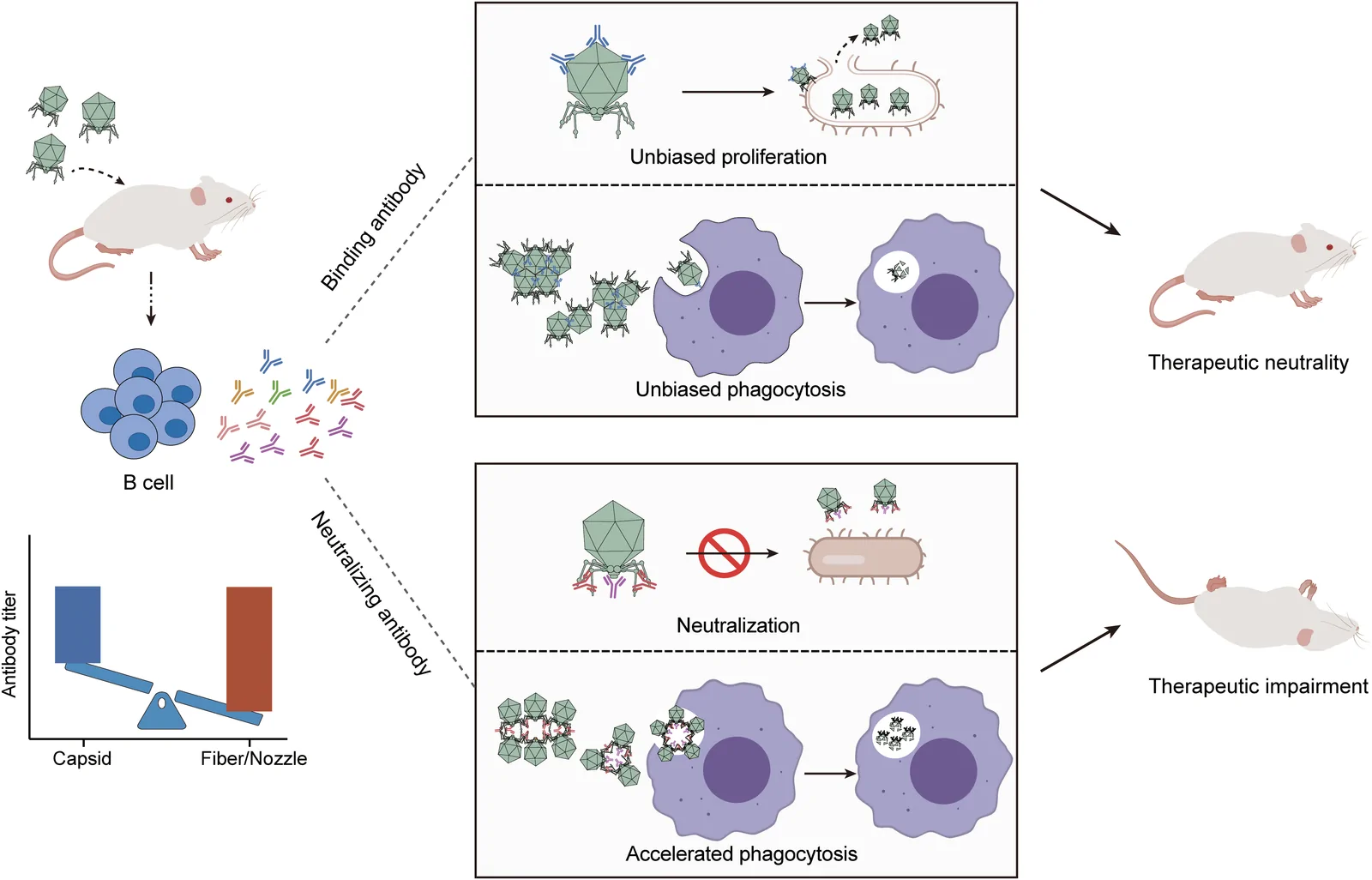
Proposed mechanisms for how phage-specific antibodies modulate the efficacy of phage therapy. Capsid-elicited binding antibodies against phage AbP20 exert minimal impact on phage proliferation. Overall, antibodies elicited by the capsid, portal, and adaptor proteins impose limited effects on phage therapeutic potency. By contrast, neutralizing antibodies targeting the fiber and nozzle proteins of AbP20 suppress phage proliferation by blocking phage-host interaction and drive the assembly of large phage aggregates that confer higher susceptibility to macrophage-mediated phagocytosis. In summary, antibodies elicited by the nozzle and fiber proteins of AbP20 ultimately impair the in vivo therapeutic efficacy of this phage.

## Discussion

Phage therapy has re-emerged as a promising approach in response to the rise of multidrug-resistant bacterial infections, demonstrating notable therapeutic efficacy in clinical and preclinical settings [3,10–12,34]. Although phage therapy has been used for more than a century [40], a widely accepted and standardized treatment protocol has yet to be established. One contributing factor is that, unlike antibiotics, phages are recognized by the host as foreign antigens and can be rapidly cleared by the immune system or neutralized by specific antibodies, thereby losing their antibacterial activity [41]. However, the impact of phage-specific antibodies on the outcome of phage therapy remains insufficiently evaluated. In the present study, we adopted podovirus AbP20, a phage that specifically infects multidrug-resistant *A. baumannii*, as a model to dissect how antibodies elicited by distinct phage structural proteins modulate phage therapy outcomes. We differentiated binding antibodies from neutralizing antibodies induced by individual structural proteins and further elucidated the underlying mechanisms by which these two antibody subsets regulate phage therapy efficacy in mouse models of *A. baumannii* infection.

Resembling other phages, repeated exposure to AbP20 induced high levels of phage-specific antibodies in mouse, accompanied by strong neutralizing activity, which ultimately led to the failure of phage therapy. As previously reported, the neutralization of phages by antibodies can significantly compromise clinical outcomes, representing a particular challenge for the development of phage therapy in the real world [15]. After administration of phage AbP20, specific neutralizing antibodies were primarily induced by its fiber and nozzle. Antibodies targeting either of these components were sufficient to cause therapeutic failure in mouse models of *A. baumannii* infection. The fiber mediates bacterial adsorption, while the nozzle is essential for DNA injection when the phage infects a bacterial cell. Although studies have reported that antibodies against phage capsid may also give rise to neutralizing effects [27], this was not observed in AbP20.

Furthermore, although residual endotoxin within the phage solution may interfere with innate immune activation and phagocytic phenotypes of macrophage, our observations with unified endotoxin dosage indicated that phage-specific antibodies influence macrophage-mediated phagocytosis and thus promote the immune clearance of phage AbP20. These observations align with previous reports showing that phage ΦX174 was cleared significantly faster from the blood of immunized mice compared to nonimmunized mice, and that immunosuppressants delayed phage clearance from the circulation [42]. Specifically, neutralizing antibodies targeting the fiber and nozzle proteins of AbP20 promoted phage phagocytosis in an Fc-receptor independent manner, whereas antibodies against the internal proteins gp35 and gp39, which are less accessible, did not showed effect on phagocytosis. Notably, antibodies against the capsid protein (gp37) also failed to enhance phagocytosis of AbP20 by macrophage in our system. This observation appears inconsistent with previous reports suggesting that Fc-mediated effector functions of phage-specific antibodies may contribute to splenic clearance through mechanisms such as antibody-dependent cellular phagocytosis [43–45]. We propose two potential mechanisms underlying this discrepancy. The first lies in differential antibody isotype profiles: mice produced relatively low levels of IgG2a and IgG2b antibodies targeting the major capsid protein (gp37) of AbP20. The second mechanism involves antibody-triggered phage aggregation, which alters the physicochemical properties of phage particles and may consequently affect macrophage phagocytic uptake efficiency. This speculative mechanism is partially supported by transmission electron microscopic observations: antibodies targeting fiber and nozzle proteins promoted the formation of large phage aggregates, in which multiple phage particles appeared to cluster with spike-like tails juxtaposed toward one another while spherical capsids faced the aggregate exterior, a spatial arrangement that may facilitate phagocytic clearance. In contrast, relatively smaller phage clusters were detected when phages were incubated with gp37-induced antibodies. These findings show that antibody-mediated phage phagocytosis serves as a critical modulator in determining the efficacy of phage therapy. Nevertheless, the native conformational state of antibody-bound phage particles, IgG subclass-specific immunization tests, as well as their correlations with host immune recognition and clearance, especially the causal relationship between the geometry of antibody-mediated aggregates and in vivo phage clearance, still require further in-depth evaluation. Given that nozzle and fiber proteins are functionally conserved among the majority of phages infecting gram-negative bacterial pathogens, systematic phenotypic and functional evaluation of phages with distinct morphological features will help clarify whether the observed antibody-mediated regulatory effects represent strain-specific responses unique to AbP20, or universally applicable to diverse therapeutic phages.

In brief, phage AbP20 structural protein-specific antibodies impair phage therapy via two synergistic, interconnected pathways: targeted infectivity neutralization and aggregation-dependent immune clearance. First, anti-gp44 fiber antibodies block phage adsorption onto host bacteria, while anti-gp40 nozzle antibodies inhibit phage DNA injection post bacterial binding; both patterns achieve phage neutralization and lowered EOP. Second, bivalent full-length antibodies trigger distinct phage aggregation patterns based on targeted protein epitopes. Phage aggregation induced by gp37-targeted antibodies exerts minor therapeutic impairment, as this aggregation pattern barely hinders phage-bacterium binding and does not facilitate macrophage phagocytic uptake. By contrast, phage clustering mediated by gp40 and gp44 antibodies (containing, in principle, tail-to-tail phage aggregates) aggravates therapeutic failure dual-modally: aggregated phage particles lose efficient bacterial adsorption capacity to amplify neutralization efficacy, and enlarged phage aggregates are preferentially engulfed and cleared by macrophages independent of Fc-mediated opsonization.

The influence of phage-specific antibodies on the outcome of phage therapy remains an emerging area where knowledge is still being accumulated. Although the animal model constructed herein fails to mimic primary phage therapy for antibody-naïve patients, it has solid clinical implications for patients undergoing repeated phage administration, as well as those with pre-existing phage-specific antibodies induced by ubiquitous environmental free phages and bacterial endogenous prophages. In addition, our findings verify that in vitro phage evolution serves as a promising strategy to evade antibody-mediated phage neutralization. Apart from adaptive evolution, other phage modification strategies including glycosylation and biomaterial encapsulation have also been documented to block the production and antigen binding of phage-elicited neutralizing antibodies [46,47]. Given that the immune responses are largely phage-specific and even phage protein-specific, it necessitates the consideration of flexible phage therapy strategy for individual patients in the absence of detailed phage-antibody interaction profiles. For instance, sequential use of phages that differ in structural proteins, especially tail components, among each other, allowing one phage may overcome phage-specific antibodies induced by others, also employing rotational dosing regimens to circumvent phage neutralization effects, and using material engineering technologies to protect phages from fast and direct recognition by the immune system. All these potential approaches must be grounded in a thorough understanding of phage–antibody interactions, including analyses at the protein level and, most probably, the individual antigenic profiles of specific phages.

## Materials and methods

### Ethics statement

All mouse experiments were conducted in an ABSL-2 lab, and all experimental methods were carried out in accordance with the regulations and guidelines set forth by the Animal Experiments Committee of the Wuhan Institute of Virology, Chinese Academy of Sciences. All experimental protocols were approved by the Animal Experiments Committee of Wuhan Institute of Virology, Chinese Academy of Sciences (WIVA41202201).

### Phage purification

Phage AbP20 was added at an MOI of 0.01 to the exponentially growing culture of *A. baumannii* strain Ab28 and cocultured for 4–6 h to allow phage proliferation. Afterwards, supernatant of the phage culture was harvested by centrifugation for 10 min at 8,000 rpm, then 1 M NaCl and 10% PEG 8,000 were added to the supernatant and stored at 4°C overnight for phage precipitation. Precipitated phage pellet collected by centrifugation at 10,000 × *g* for 1 h was resuspended in PBS and mixed with an equal volume of chloroform. Following centrifugation at 3,500 rpm, phage was collected from the upper aqueous phase and subjected to CsCl density gradient centrifugation at 180,000 × *g* for 2 h to remove lipopolysaccharide and bacterial contaminants. Phage preparations were filtered through a 0.22 μm filter and stored at 4 °C after extensive dialysis. Endotoxin concentration was determined by the endpoint chromogenic method using the ToxinSensor Chromogenic LAL Endotoxin Assay Kit (GenScript Biotech Corp., USA) according to manufacturer’s instructions. Phage titer was determined by the plaque assay described below.

### Phage plaque assay

Phage quantification was performed using the double-agar overlay plaque assay. Briefly, *A. baumannii* Ab28 was overlaid onto an LB (lysogeny broth) solid agar base in 0.7% soft agar. Serially diluted phage AbP20 was then spotted onto the double-layer plates. After incubating at 37 °C for 6 h, plaques were counted and phage titer was calculated.

### Negative-stain transmission electron microscopy

A phage suspension at a concentration of 10¹¹ PFU/ml was applied to a carbon-coated copper grid for 1 min, followed by negative staining with 2.0% phosphotungstic acid (pH 7.0) for 2 min. Electron images were recorded on a Talos L120C transmission electron microscope.

### Bactericidal activity of AbP20 against *A. baumannii* Ab28

To test the inhibitory effects of AbP20 on the growth of *A. baumannii*, exponential cultures of Ab28 were diluted with LB to an OD_600_ of 0.4 and incubated with different MOIs (0, 0.01, 0.1, 1, and 10) of AbP20 at 37 °C with shaking at 180 rpm. The turbidity of bacterial culture at 600 nm was monitored every 30 min for 12 h. To determine the bactericidal activity of phage AbP20 against Ab28, log‑phase culture of Ab28 was cultured in LB at 37 °C with shaking at 200 rpm in the presence of AbP20 an MOI of 1. Viable bacteria were quantified by enumerating the CFU at 0, 1, 2, 4, and 6 h of coculture. All experiments were carried out in triplicate.

### Purification of phage structural proteins

Genes coding for each individual structural protein of AbP20, i.e., gp35, gp37, gp39, gp40, and gp44, were cloned into the pET-28a(+) expression vector with a C‑terminal 6 × His tag using gene-specific primers (S1 Table) and transformed into *E. coli* BL21(DE3) for expression. Recombinant proteins were purified through Ni‑NTA affinity chromatography and dialyzed against PBS. Protein concentration and purity were assessed using NanoDrop spectrophotometry and SDS‑PAGE.

### Mouse experiments

To assess the safety of AbP20, 6–8-week-old female BALB/c mice were intraperitoneally administrated with 100 μL of 1 × 10^10^ PFU/mL AbP20 daily for 7 consecutive days. Body weights were monitored daily for 10 days. One day after the last phage administration, peripheral blood from each mouse was collected for hematological analysis, the heart, liver, spleen, lungs, kidneys, and brain were harvested for histopathological examination.

To evaluate the efficacy of phage therapy in the absence of phage-specific antibodies, 6–8-week-old female BALB/c mice were infected intraperitoneally with 200 μL of 1 × 10⁹ CFU/mL *A. baumannii* Ab28. At 1- and 8-h post-infection, mice were intraperitoneally administrated with either 30 mg/kg apramycin, 2 × 10^8^ PFU/mouse phage AbP20, 2 × 10^9^ PFU/mouse AbP20, or an equal volume of PBS buffer. Survival in each group was monitored every 12 h for 6 days (*n* = 5). At 10 h post infection, peripheral blood was collected from each group for hematological analysis (*n* = 3). At 24 h post infection, the liver, kidney, and spleen were harvested from each group for CFU enumeration (*n* = 5).

To evaluate the efficacy of phage therapy after repeated exposure to AbP20, 6–8-week-old female BALB/c mice were intraperitoneally administrated with either 100 μL of AbP20 (1 × 10^10^ PFU/mL) or an equal volume of PBS once daily for 7 consecutive days. Four weeks after the last stimulation, mice were intraperitoneally challenged with 2 × 10^8^ CFU/mouse of *A. baumannii* Ab28 and then received 2 × 10^9^ PFU/mouse of AbP20 intraperitoneally at 1- and 8-h post‑infection. Survival was monitored every 12 h for 6 days (*n* = 6). At 24 h post infection, the liver, kidney, and spleen were harvested from each group for CFU enumeration (*n* = 6). Ten days before infection, serum from each group was collected via retro‑orbital bleeding for antibody titer determination.

To evaluate the efficacy of phage therapy in the presence of phage-specific antibodies, 6–8-week-old female BALB/c mice were immunized intramuscularly with either 5 × 10^10^ PFU/mouse of AbP20, 10 μg aluminum adjuvanted each of gp35, gp37, gp39, gp40, and gp44, or an equal volume of PBS with aluminum adjuvant three times at 2-week intervals. Two weeks after the last boost, mice were intraperitoneally challenged with 2 × 10^8^ CFU/mouse of *A. baumannii* Ab28 and then received 2 × 10^9^ PFU/mouse of AbP20 intraperitoneally at 1- and 8-h post‑infection. Survival was monitored every 12 h for 6 days (*n* = 6). At 24 h post infection, the liver, kidney, and spleen were harvested from each group for CFU enumeration (*n* = 4). Three days before infection, serum from each group was collected via retro‑orbital bleeding for antibody titer determination.

To determine phage clearance in spleen with the presence of phage-specific antibodies, 6–8-week-old female BALB/c mice were intramuscularly immunized with either 5 × 10^10^ PFU/mouse of AbP20, 10 μg aluminum adjuvanted each of gp35, gp37, gp39, gp40, and gp44, or an equal volume of PBS with aluminum adjuvant three times at 2-week intervals (*n* = 3). Two weeks after the last boost, mice were intraperitoneally administered with 5 × 10^9^ PFU/mouse AbP20 or AbP20M. At 1-, 3-, 6-, and 12-h post phage administration, spleens were harvested, homogenized, and centrifuged. The titer of viable phage in the supernatant was then determined using the double‑layer plaque assay.

To evaluate the therapeutic efficacy of the mutant phage AbP20M in mice pre-exposed to AbP20, 6–8-week-old female BALB/c mice were immunized intramuscularly with 5 × 10^10^ PFU/mouse of AbP20. Two weeks after the last boost, mice were intraperitoneally challenged with 2 × 10⁸ CFU/mouse of *A. baumannii* Ab28 and then received intraperitoneal administration of AbP20M or the wild-type phage AbP20 at 2 × 10⁹ PFU/mouse (MOI = 10) or 2 × 10^10^ PFU/mouse (MOI = 100) at 1- and 8-h post‑infection. Survival was monitored every 12 h for 6 days (n = 6). At 24 h post‑infection, the liver, kidney, and spleen were harvested from each group for CFU enumeration (*n* = 4). Three days before infection, serum from each group was collected via retro‑orbital bleeding for determination of antibody titers and neutralizing activity.

### Blood routine examination

Blood collected in microcentrifuge tubes containing EDTA-K2 was gently mixed 8–10 times by inversion to ensure homogeneity. Hematological analysis was subsequently performed using an automated hematology analyzer (HEMAVET 950FS).

### Histopathological examination

Mouse tissues were collected, fixed in 4% paraformaldehyde, and embedded in paraffin. The paraffin-embedded tissues were then sectioned at 5 μm thickness and stained with hematoxylin and eosin for histological examination. The stained sections were then observed and photographed using a digital image analysis platform 3DHISTECH (Hungary).

### Purification of IgG from serum

Serum was diluted 1:5 with Binding/Wash buffer (20 mM disodium hydrogen phosphate, 0.15 M sodium chloride, pH 7.0). A 1.5 mL aliquot of Protein G resin was added to a chromatography column preloaded with 1.5 mL of Binding/Wash buffer. Before sample loading, the column was equilibrated with 5 mL of Binding/Wash buffer. The diluted serum was then loaded and allowed to flow out slowly. After washing with 30 mL of Binding/Wash buffer, bound IgG was eluted using Elution buffer (0.1 M glycine, pH 2.5). One‑10th volume of Neutralization buffer (1 M Tris‑HCl, pH 8.5) was added to the eluate to adjust the pH to 7.4. The antibody concentration was determined, and the purified IgG was aliquoted and stored at −80 °C until use.

### Preparation of IgG Fab fragments

The prepared IgG was dialyzed against 0.01 M PBS (pH 7.4) for 3 h, and the antibody concentration was adjusted to 1 mg/mL. Papain powder was dissolved in water to prepare a 10 mg/mL stock solution, which was filtered through a 0.22 μm filter and stored at 4 °C. The enzyme was diluted to 1 mg/mL in a solution containing 1.1 mM EDTA, 0.067 mM β‑mercaptoethanol, and 5.5 mM cysteine‑HCl, and then incubated for 30 min for activation. Papain and the antibody solution were mixed at a ratio of 1:10 and incubated at 37 °C for 12 h. The reaction was stopped by adding 33 mM N‑ethylmaleimide. The reaction product was ultrafiltrated using a 30 kDa ultrafiltration tube to remove papain and exchange the buffer to 0.01 M PBS (pH 7.4). The digested product was passed through a Protein G resin, and the flow‑through fraction was collected to remove Fc fragments. Each fraction was verified by SDS‑PAGE.

### Serum neutralization assay

Ten μL of 2 × 10^10^ PFU/mL phage AbP20 was mixed with 2 μL of varied immune serum or purified IgG, and 88 μL of PBS and then incubated at 37 °C for 1 h. The phage‑serum mixture was then serially diluted in PBS and titrated using the double‑layer plaque assay. The phage neutralization efficacy of each serum was presented as efficiency of plating (EOP) that calculated as the ratio of the phage titer in each treatment to the titer in the serum‑free control.

### ELISA assay

Phage AbP20 or individual phage structural proteins were diluted in 100 μL of coating buffer (bicarbonate‑carbonate, pH 9.6) and coated at 2 × 10⁹ PFU/well of phage or 500 ng/well of protein in 96-well plates (Corning) overnight at 4 °C. After washing four times with 300 μL of PBST (PBS containing 0.05% Tween‑20), plates were incubated with 5% skim milk in TBST for 2 h at 37 °C. Subsequently, 100 μL of diluted immune serum was added and incubated at 37 °C for 2 h. Following four washes with 300 μL PBST, plates were supplemented with 100 μL/well of HRP‑conjugated goat anti‑mouse IgG or its subtypes (1:10,000 diluted) and incubated at 37 °C for 1 h. After washing another four times with PBST, each well was incubated with 100 μL of 3,3′,5,5′‑tetramethylbenzidine substrate for 15 min at room temperature and then supplemented with 2 M H2_2_SO_4_. The endpoint titer of immune serum was defined as the highest dilution fold that yielded an absorbance at 450 nm more than 2.1‑fold of the mean value from naïve mouse serum-treated groups.

### Determination of the neutralization stage

To determine the effect of each structural protein-elicited serum on phage adsorption, serum diluted 100‑fold were incubated with 10⁸ PFU of AbP20 at 37 °C for 1 h, followed by incubation with 10⁸ CFU/mL of Ab28 at 4 °C for 2 h. After five washes with PBS, samples were analyzed by Western blotting. Gray values were calculated using ImageJ software. To determine the effect of serum against different components of AbP20 on phage DNA injection, sera diluted 100‑fold were incubated with 10⁸ PFU of AbP20 at 37 °C for 1 h, followed by incubation with 10⁸ CFU/mL of Ab28 at 37 °C for 20 min. After five washes with PBS, the copies were determined by qPCR as described below, and the inhibition rate was calculated. To determine the effect of serum against different components of AbP20 on phage release, sera diluted 100‑fold were incubated with 10⁸ PFU of AbP20 at 37 °C for 1 h, followed by incubation with 10⁸ CFU/mL of Ab28 at 37 °C for 2 h. After centrifugation, the supernatant was collected, and phage titers were determined using the double‑layer agar plate method.

### Phage nucleic acid extraction and qPCR detection

Phage nucleic acids were extracted from bacterial samples using the FineMag Rapid Magnetic Bead Method Virus DNA/RNA Extraction Kit (GENFINE) according to the manufacturer’s instructions. Quantitative polymerase chain reaction (qPCR) was performed with the Hieff SYBR Green qPCR Master Mix (YEASEN). All qPCR assays were carried out on a Bio-Rad CFX96 instrument (Bio-Rad). The threshold cycle (Ct) value for each sample was determined using Bio-Rad CFX Manager 3.1 software. The RNA polymerase β subunit gene fragment of Ab28 was used as an internal control. The qPCR primer sequences are listed in S1 Table.

### Polyclonal rabbit anti-gp37 antibodies

The gp37 protein was mixed with an aqueous rapid immunoadjuvant for rabbit immunization. Following the immunization, antiserum was collected and subjected to titer determination. Subsequently, 1 mg of the purified protein was covalently conjugated to a cyanogen bromide-activated Sepharose 4B column to prepare an affinity purification column and incubated with the antiserum overnight. After washing with HCl (pH 5.0) to remove nonspecific antibodies, column was eluted with 0.15 M Glycine-HCl buffer (pH 2.5), and the passthrough solution was immediately neutralized with 10 × PBS. The concentration of the purified antibody was measured using the Bradford assay, followed by further titer validation to ensure that their binding activity and specificity met experimental requirements.

### Detection of phage phagocytosis in cell

RAW264.7 cells were seeded in 24-well plates at a density of 3 × 10^5^ cells per well for 12 h, after replacing the medium with DMEM containing 2% FBS, each well was supplemented with 10 μL of 1 × 10^10^ PFU AbP20, 85 μL of PBS, and 5 μL of varied immune sera, purified IgGs or their Fab fragments, and then incubated for 6 h at 37 °C with 5% CO_2_. Following incubation, the cells were washed with PBS (pH 7.4) and lysed by RIPA lysis buffer (Beyotime) supplemented with protease inhibitors (Roche). Cell lysates were then mixed with 5 × loading buffer, boiled at 100 °C for 10 min, and then analyzed by SDS‑PAGE. Resolved protein bands were transferred onto a polyvinylidene fluoride membrane (Millipore) and blocked with 5% skimmed milk in PBST 1 h at room temperature, followed by overnight incubation with rabbit polyclonal anti-gp37 antibodies at 4 °C. After four washes with PBST, the membrane was incubated with horseradish peroxidase-conjugated anti‑rabbit IgG (Proteintech). Protein bands were visualized using an enhanced chemiluminescence kit (Millipore). The band intensity of gp37 from each group was quantified by Image J and compared to that of the PBS-immunized group (Vehicle). All experiments were carried out by triplicates taking GAPDH as reference.

### Fc receptor blocking assay

RAW264.7 macrophages were seeded in 24-well plates at a density of 3 × 10^5^ cells per well and cultured for 12 h. Afterwards, the culture medium was replaced with DMEM supplemented with 2% FBS, followed by the addition of MonoZero Mouse Fc Blocking Reagent (Proteintech) at a dosage of 0.5 μg per 100 μL cell suspension. Following gentle pipette mixing, cells were incubated at 37 °C for 20 min. The premixed phage-serum sample was subsequently added to each well, and co-cultured with cells at 37 °C for 6 h. Finally, cellular samples were harvested for subsequent western blot analysis.

### Immunofluorescence assay

RAW264.7 cells were seeded in 24-well plates at 2 × 10^5^ cells per well for 12 h, after replacing the medium with DMEM containing 2% FBS, each well was supplemented with 10 μL of 1 × 10^10^ PFU AbP20, 85 μL of PBS, and 5 μL of varied immune serum and then incubated for 6 h at 37 °C with 5% CO_2_. Subsequently, wells were washed five times with PBS to remove uninternalized phage and then fixed with 4% paraformaldehyde for 15 min. Following three times washing with PBS, cells were permeabilized with 0.1% Triton X-100 for 10 min and followed by another three PBS washes before blocking with blocking solution (Beyotime) for 30 min. Subsequently, cells were incubated overnight at 4 °C with a rabbit polyclonal anti-gp37 antibodies (1:200 diluted). After three washes with PBS, cells were incubated for 1 h at room temperature in dark with a CoraLite488-conjugated Goat Anti-Mouse IgG(H + L) secondary antibody (1:200 diluted). Following three additional washes with PBS, the cytoskeleton was stained using ActinRed 555 ReadyProbes Reagent (Thermo Fisher) for 30 min at room temperature. Finally, after three further PBS washes, cells were stained with 4′,6-diamidino-2-phenylindole (DAPI; Leagene) at a concentration of 1 μg/ml for 10 min at room temperature. Cells were then mounted with Antifade Mounting Medium (Beyotime) and imaged using a Leica STELLARIS 8 WLL confocal fluorescence microscope. The mean fluorescence intensity in each treatment was calculated by LAS X software from six distinct regions and compared to that of the group treated with PBS-immunized serum (Vehicle).

### Detection of serum-mediated phage aggregation

To detect the size distribution of phage aggregates, 20 μL of 1 × 10^10^ PFU AbP20 was mixed with 170 μL of PBS and 10 μL of varied immune sera, purified IgGs, or their Fab fragments and incubated at 37 °C for 1 h. Subsequently, the diameter distribution of phage aggregates was measured by dynamic light scattering using a Zetasizer Nano ZS (Malvern Panalytical, UK). To capture the image of phage aggregates, 10 μL of 1 × 10^10^ PFU AbP20 was incubated with 88 μL of PBS and 2 μL of varied immune serum or IgGs at 37 °C for 1 h. Samples were then imaged by transmission electron microscopy following the previously described protocol.

### In vitro phage evolution

AbP20-immune serum was diluted to a starting titer of 1:800, pre-incubated with phage AbP20 for 30 min, and subsequently co-cultured with *A. baumannii* strain Ab28. Following 6 h of co-incubation, culture supernatants were harvested via centrifugation. After gradient dilution and plaque purification, 10 independent plaques were randomly picked for serial passaging per selection round. To impose progressive immune selection pressure, the serum dilution ratio was reduced by 100-fold per round (from 1:800 sequentially to 1:700, 1:600 and ultimately 1:100), and the 1:100 serum dilution group was subjected to triple independent screening. Starting from the 4th selection round, efficiency of plating (EOP) assays were performed to assess the neutralization escape capacity of evolved phage variants. Post-selection phages were purified and amplified from single plaques in each round. The full-length coding sequences of gp40 and gp44 were amplified by PCR and subjected to sequencing to screen for mutation sites.

### Statistical analysis

Data were presented as mean ± SD. Statistical analysis was performed using GraphPad Prism (GraphPad Software). Statistical significance was calculated using a two-tailed Student *t* test. Survival curves were analyzed with Log-rank (Mantel-Cox) test by GraphPad Prism (GraphPad Software). ns: not significant, *p < 0.05, **p < 0.01, ***p < 0.001, and ****p < 0.0001.

## Supporting information

S1 Fig Activity and genome characterization of phage AbP20. (**A**) Bactericidal activity of AbP20 against *A. baumannii* Ab28. Exponential cultures of Ab28 were cultured in LB at 37 °C with shaking at 200 rpm in the presence of AbP20 at varied MOIs. Viable bacteria were quantified by enumerating the CFU at 0, 1, 2, 4, and 6 h of coculture. Dashed line indicates the limit of detection. (**B**) Genomic annotation of AbP20. The AbP20 genome was sequenced, and all predicted open reading frames are labeled. The data underlying this figure can be found in S1 Data.
(TIF)

S2 Fig Imbalance in peripheral blood cells is mitigated by high-dose AbP20 treatment. Peripheral blood counts of total neutrophils, monocytes, and platelets were assessed in AbP20-treated group. According to the manufacturer’s specifications, all measured values were determined to be within normal reference ranges. The data underlying this figure can be found in S1 Data.
(TIF)

S3 Fig Safety evaluation of phage AbP20. (**A**) Body weight changes in mice after intraperitoneal administration of 1 × 10^10^ PFU/mouse AbP20 daily for 7 consecutive days. Body weight was measured daily for 10 days. (**B**) Total white blood cell, neutrophil, lymphocyte, monocyte counts in peripheral blood from AbP20-treated mice. All values were within the normal reference range as defined by the assay manufacturer’s instructions. Dashed lines indicate the healthy range. (**C**) Histopathological evaluation. Organs were collected from mice 1 day after the final AbP20 treatment and assessed by hematoxylin and eosin staining. The data underlying this figure can be found in S1 Data.
(TIF)

S4 Fig Purification and immunogenicity evaluation of AbP20 structural proteins. (**A**) Heterologous expression of AbP20 structural proteins in *E. coli*. (**B**) Immunogenicity of AbP20 and its structural proteins. IgG titers from each immune serum (Serum) against AbP20 or each of its structural protein (Antigen) were determined by ELISA. (**C-F**) The titers of IgG subclasses in each immune serum against AbP20 or each structural protein (antigen) were determined by ELISA. Dashed line indicates the limit of detection. The data underlying this figure can be found in S1 Data. The uncropped gels can be found in S1 Raw Images.
(TIF)

S5 Fig Effects of the serum from varied groups on the infective activity of AbP20. (**A**) Schematic diagram of the neutralization assay. (**B**) Infective activity of AbP20 in the presence of varied immune serum. Phage AbP20 was incubated with varied immune serum diluted 50–800 folds at 37 °C for 1 h. The double-layer plaque assay was then used to evaluate the infective activity of AbP20. (**C**) Effect of serum against different components of AbP20 on phage adsorption. Serum diluted 100‑fold was incubated with 10⁸ PFU of AbP20 at 37 °C for 1 h, followed by incubation with 10⁸ CFU/mL of Ab28 at 4 °C for 2 h. After washing with PBS, the samples were analyzed by Western blotting. The uncropped blots and gels can be found in S1 Raw Images.
(TIF)

S6 Fig AbP20 immune serum increases macrophage-mediated phage phagocytosis. (**A**) IFA of AbP20 phagocytosis by macrophages after 6 h incubation with AbP20 immune serum. Phage AbP20 is labeled by rabbit anti-gp37 antibody and CoraLite 488 dye (green), β-actin stained with ActinRed 555 (red), and the nucleus stained with DIPA (blue). Scale bar: 20 μm. (**B**) Quantitative analysis of the mean fluorescence intensity performed using LAS X software, with measurements taken from six distinct regions for statistical analysis. The data underlying this figure can be found in S1 Data.
(TIF)

S7 Fig Purified IgGs targeting AbP20, gp40, gp44, and gp37 retain strong neutralizing activity and promote phage phagocytosis. (**A**) Immunogenicity of purified antibodies. Sera were purified using Protein G resin to obtain purified IgG antibodies. The titers of the purified IgGs targeting each structural protein against AbP20 were determined by ELISA. (**B**) Effects of varied IgG on the efficiency of plating (EOP) of phage AbP20. (**C**) The effect of purified IgGs on AbP20 phagocytosis was assessed by western blot. Intracellular phages were detected using rabbit anti‑gp37 antibody, with Tubulin serving as a reference. Band intensities of gp37 from three independent Western blots were analyzed by Image J software and compared with those of the PBS‑treated control group. Dashed line indicates the limit of detection. Unpaired Student’s t tests. ns: not significant, *p < 0.05 and **p < 0.01. The data underlying this figure can be found in S1 Data. The uncropped blots can be found in S1 Raw Images.
(TIF)

S8 Fig Different IgG antibodies induce aggregation of phages. Representative images of AbP20 aggregates in the presence of varied IgGs. Model manners of phage conjugation under each condition are presented above each image. Scalar bar: 100 nm.
(TIF)

S9 Fig AbP20M mirrors AbP20 in lytic curve, phagocytosis, and in vivo clearance. (**A**) Inhibitory effects of AbP20M on the growth of *A. baumannii* 28. Exponential cultures of Ab28 were diluted with LB to an OD600 of 0.4 and incubated with different MOIs (0, 0.01, 0.1, 1, and 10) of AbP20 at 37 °C with shaking at 180 rpm. The turbidity of bacterial culture at 600 nm was monitored every 30 min for 12 h. (**B**) Phagocytosis of AbP20M by macrophages was detected by western blot. Intracellular phages were detected using a rabbit anti‑gp37 antibody, with tubulin serving as a reference. Band intensities of gp37 from three independent Western blots were analyzed using ImageJ software. (**C**) Evaluation of the effects of antibodies on the clearance of phage AbP20M in vivo. Group immunized with PBS served as control (Vehicle). All mice received a single dose of AbP20 intraperitoneally 14 days post the last boost. Spleens were collected at 1-, 3-, 6-, and 12-h post-injection for phage titer determination (*n* = 3). Unpaired Student’s *t* tests. ns: not significant. The data underlying this figure can be found in S1 Data. The uncropped blots can be found in S1 Raw Images.
(TIF)

S1 Table Primers used in this study. (DOCX)

S1 Raw Images All the uncropped western blot and SDS-PAGE images contained in the manuscript. Uncropped western blot images corresponding to Figs 3B, 3D, 5A, 5B, 5E, 5F, 5G, 5H, 6F–6H, S4A, S5C, S7C, and S9B.
(PDF)

S1 Data The numerical data underlying Figs 1B, 1D–1G, 1I–1L, 2C–2J, 3C, 3E, 3G, 3I, 4A, 5A, 5C–5H, 6C, 6E–6H, 6J–6M, S1A, S2, S3A, S3B, S4B–S4F, S6B, S7C, and S9A–S9C. (XLSX)

## Abbreviations

ADCP: antibody-dependent cellular phagocytosis
EOP: efficiency of plating
IFA: immunofluorescence assays
MHC: major histocompatibility complex
ORFs: open reading frames
qPCR: quantitative polymerase chain reaction
TCRs: T cell receptors
Wb: Western blotting.
