# Supplementary material for: Antibodies targeting phage fiber and nozzle proteins impair Acinetobacter baumannii phage therapy by blocking infection and promoting immune clearance
Source: PLoS Biol. 2026 Sep 22;24(9):e3004009. doi: 10.1371/journal.pbio.3004009 (PMC13623143; doi:10.1371/journal.pbio.3004009)
Supplement: Supplementary file 1 [file pbio.3004009.s011.pdf]

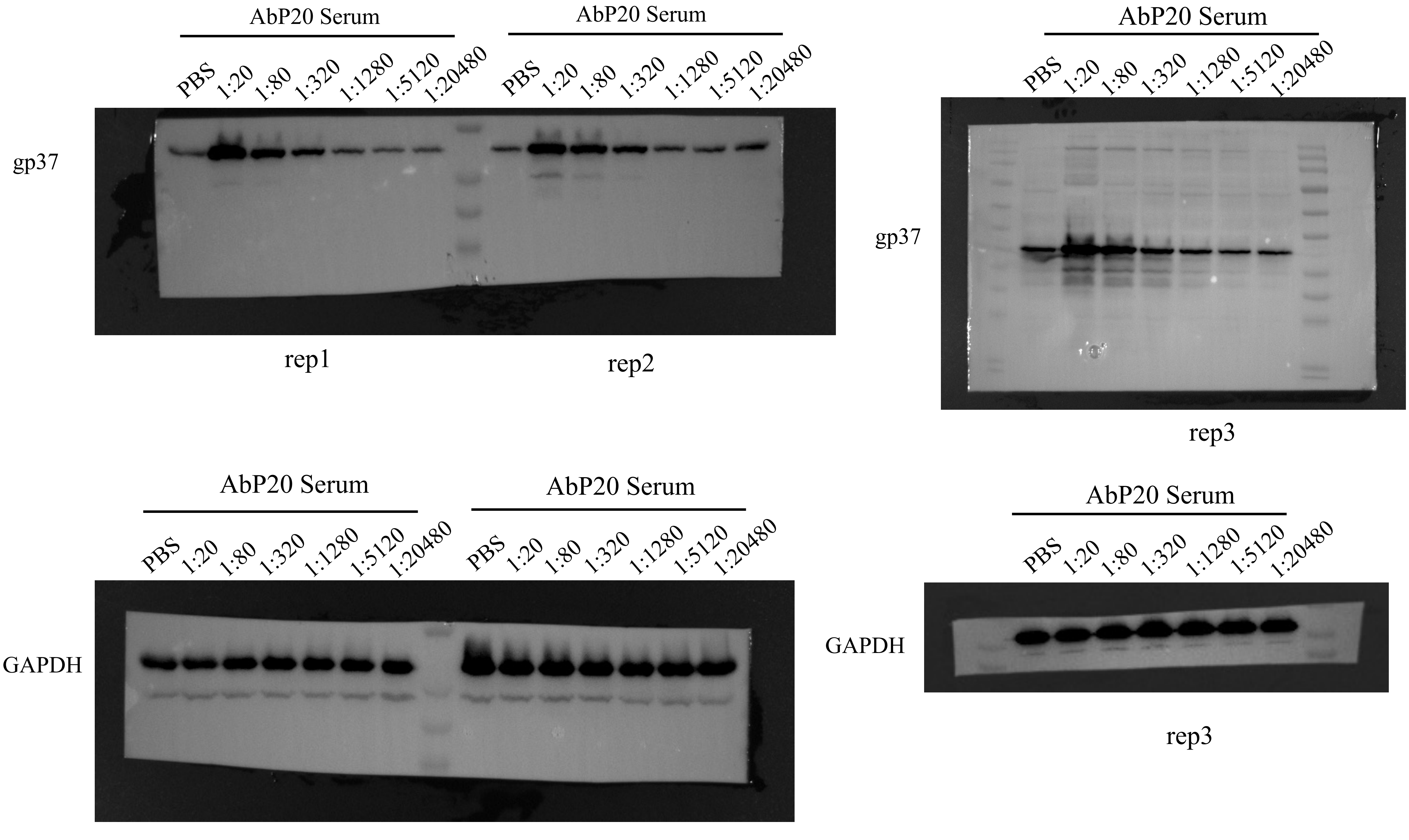

Fig3B Western blot

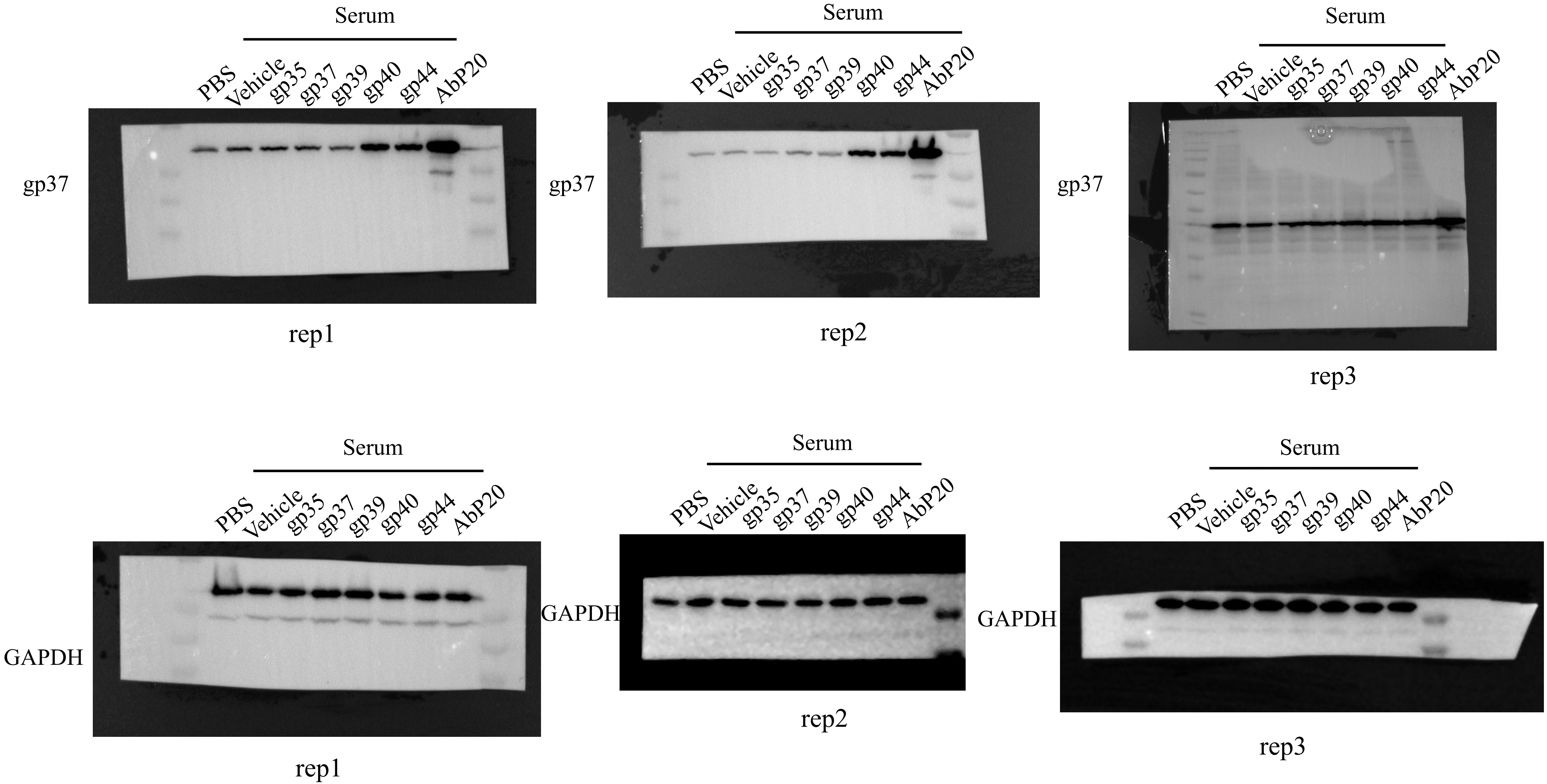

Fig3D Western blot

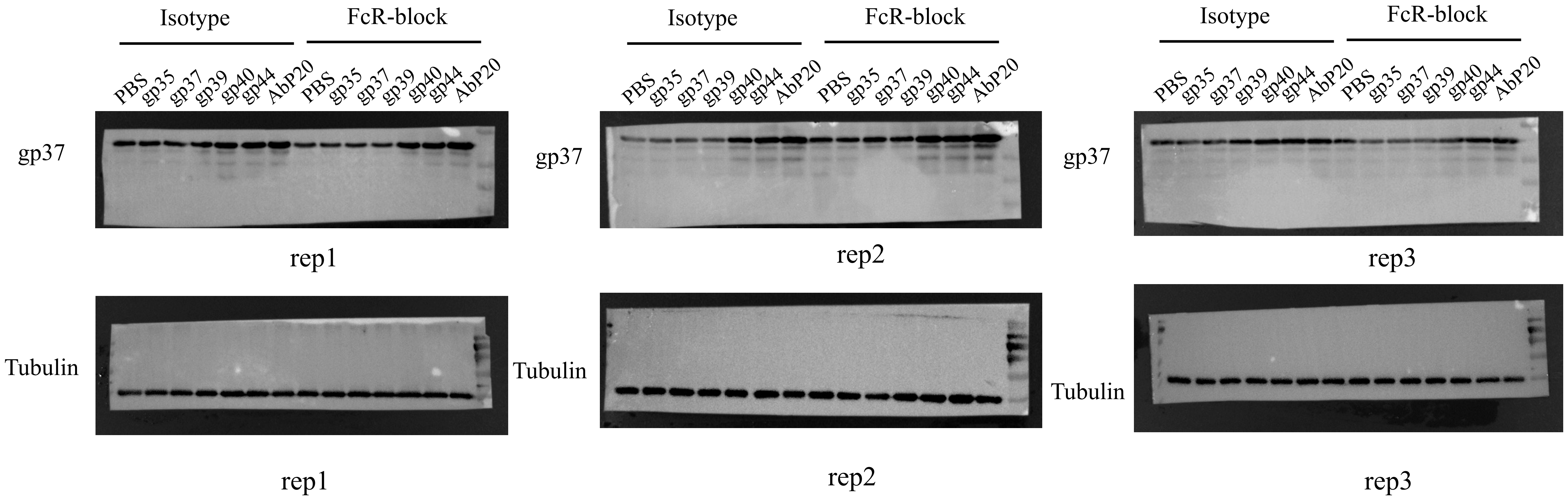

Fig5A Western blot

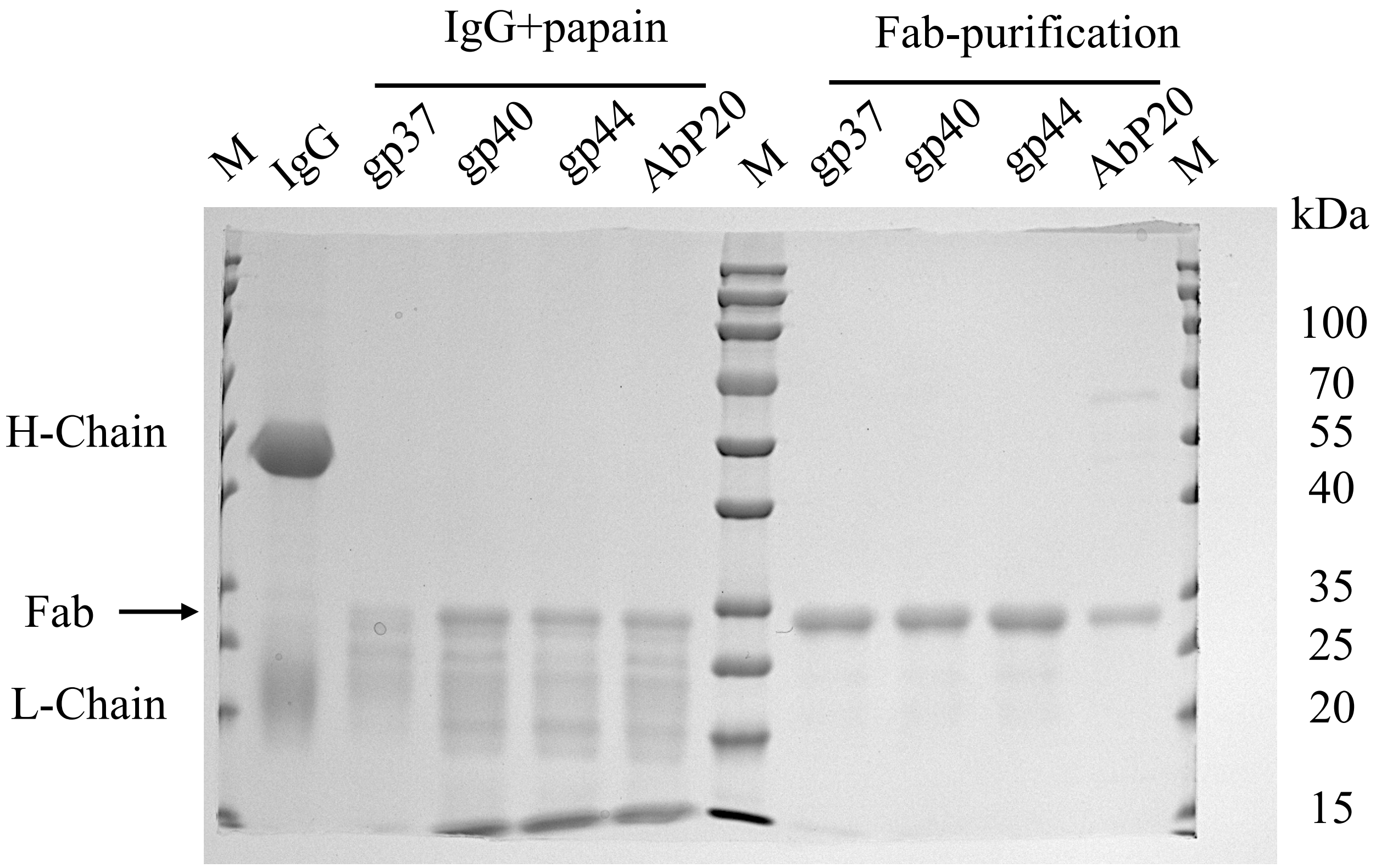

Fig5B SDS-PAGE

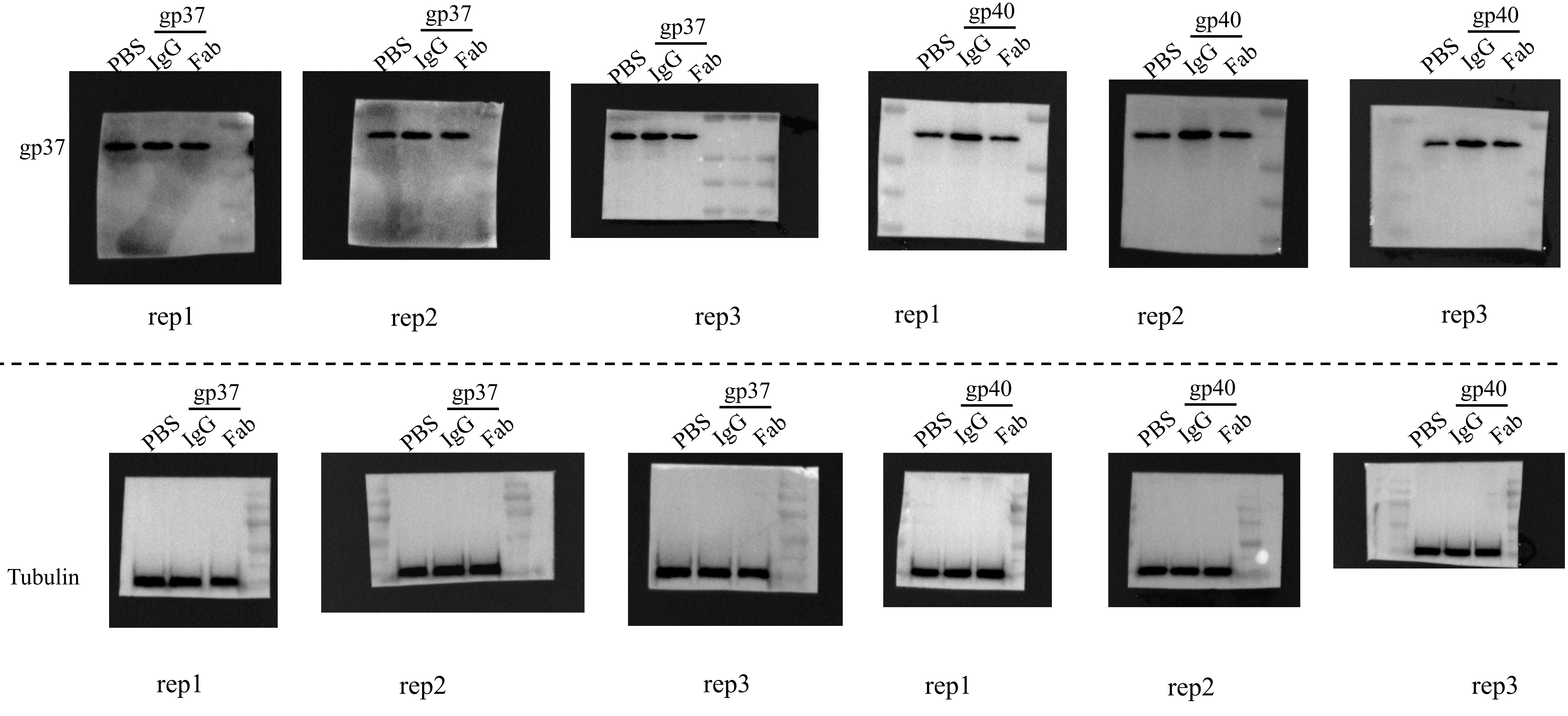

Fig5E-F Western blot

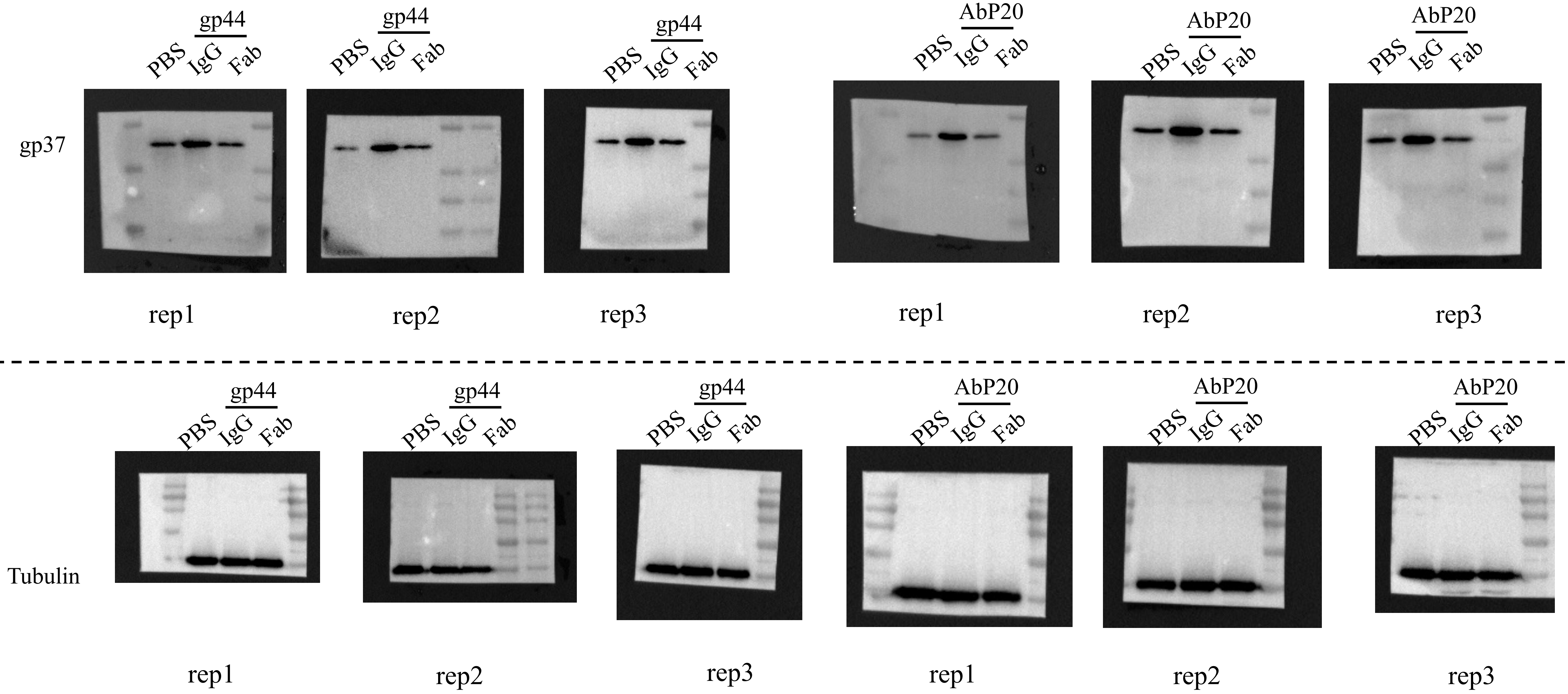

Fig5G-H Western blot

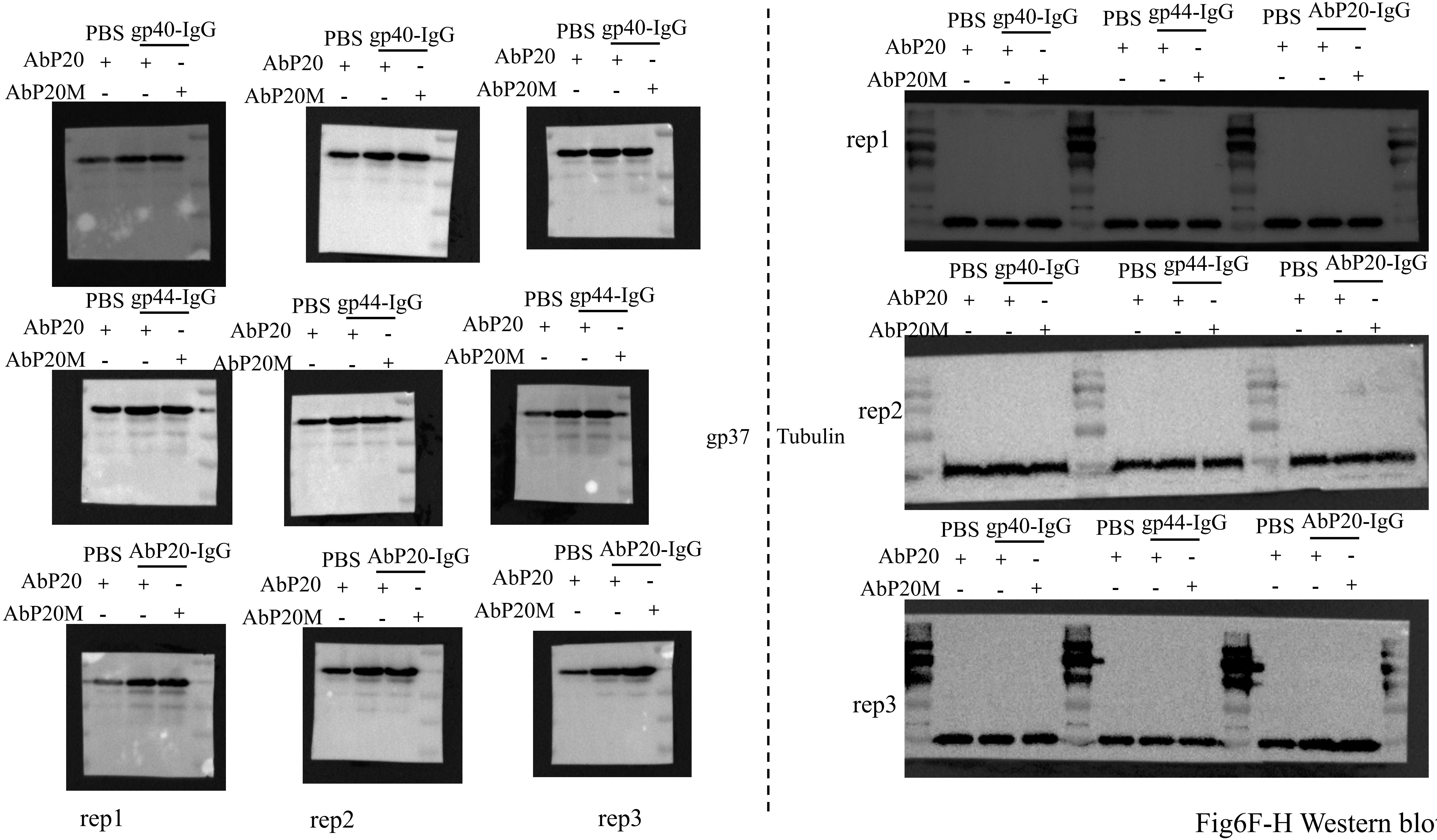

Fig6F-H Western blot

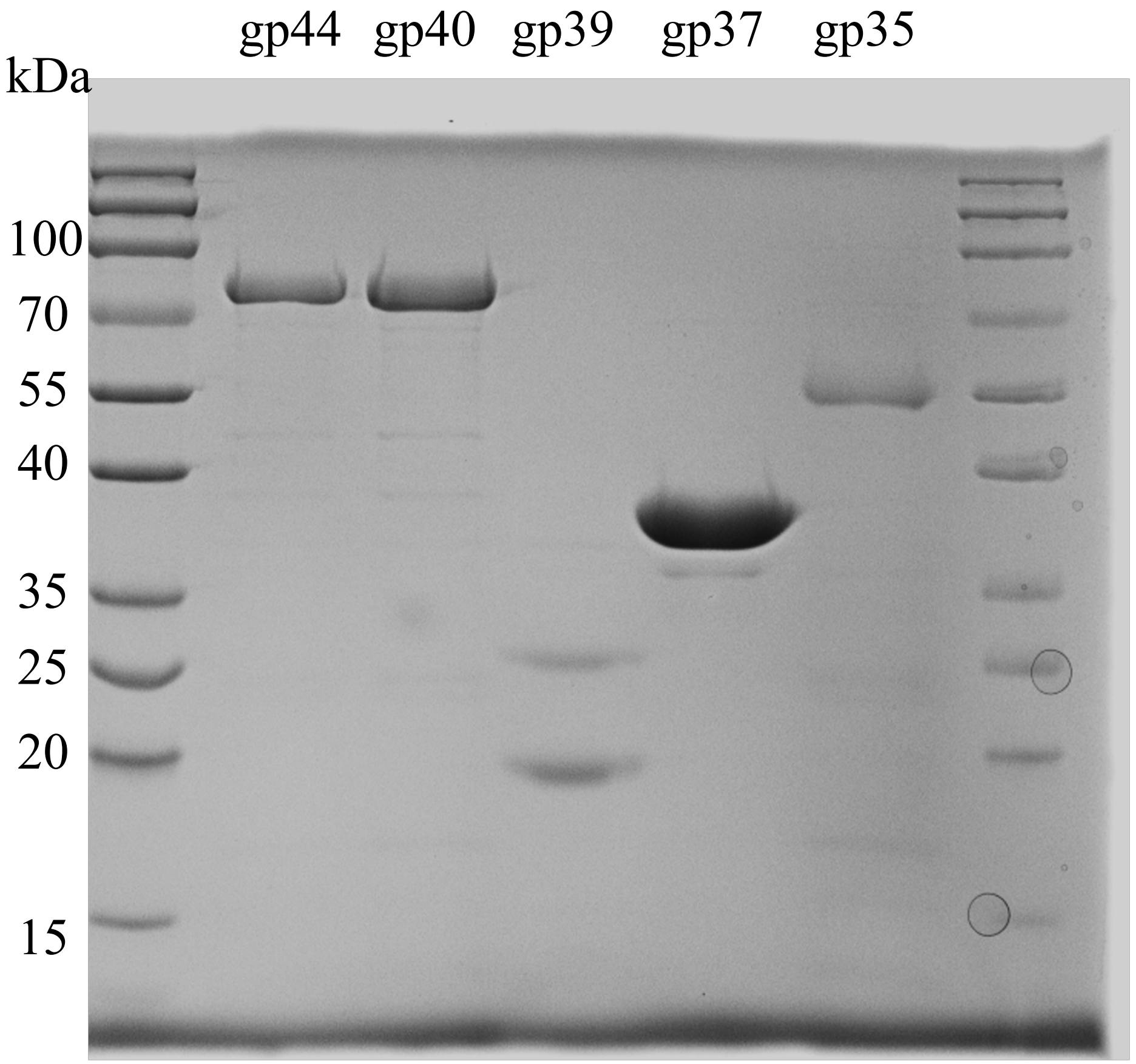

FigS4A SDS-PAGE

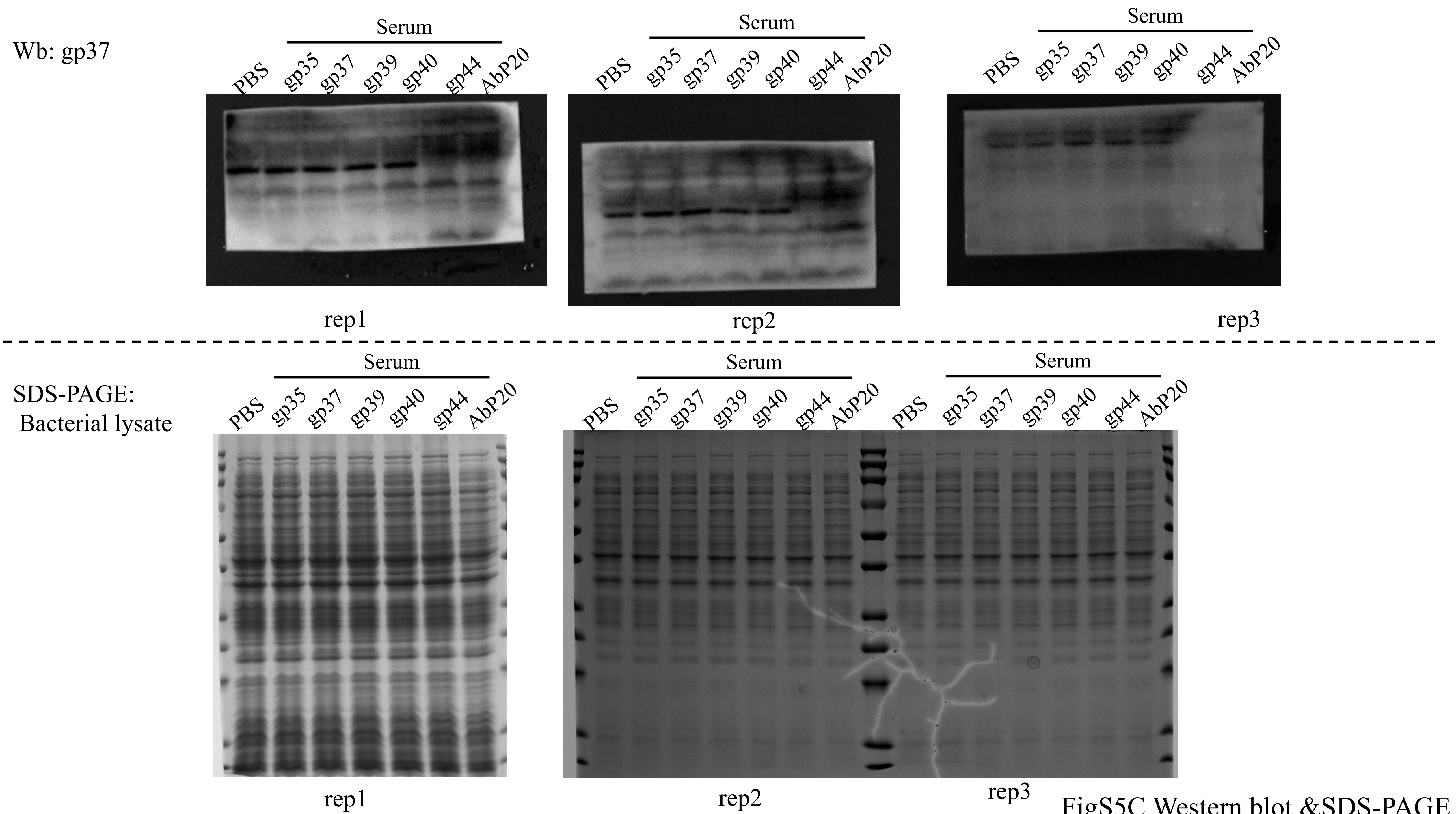

FigS5C Western blot &SDS-PAGE

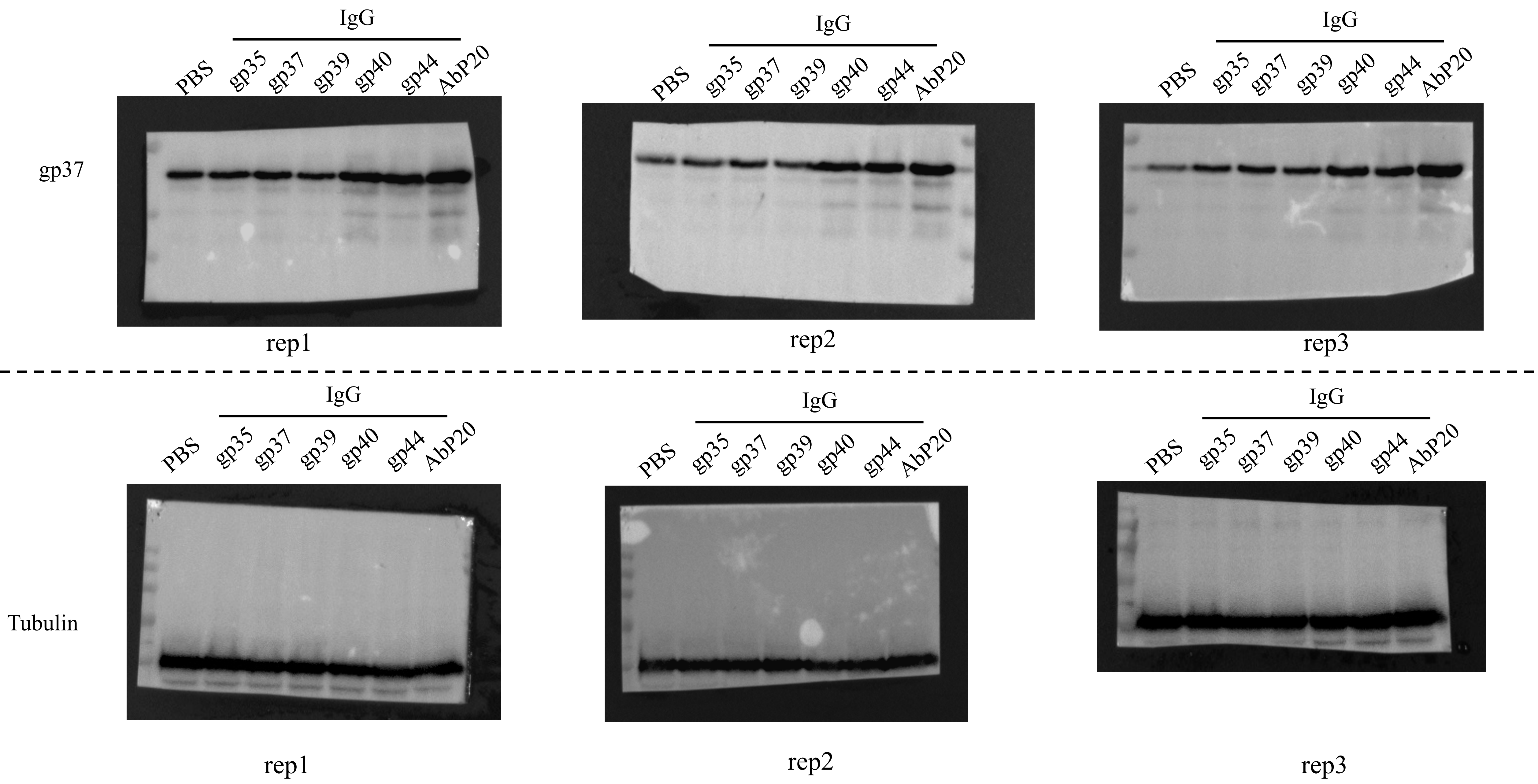

FigS7C Western blot

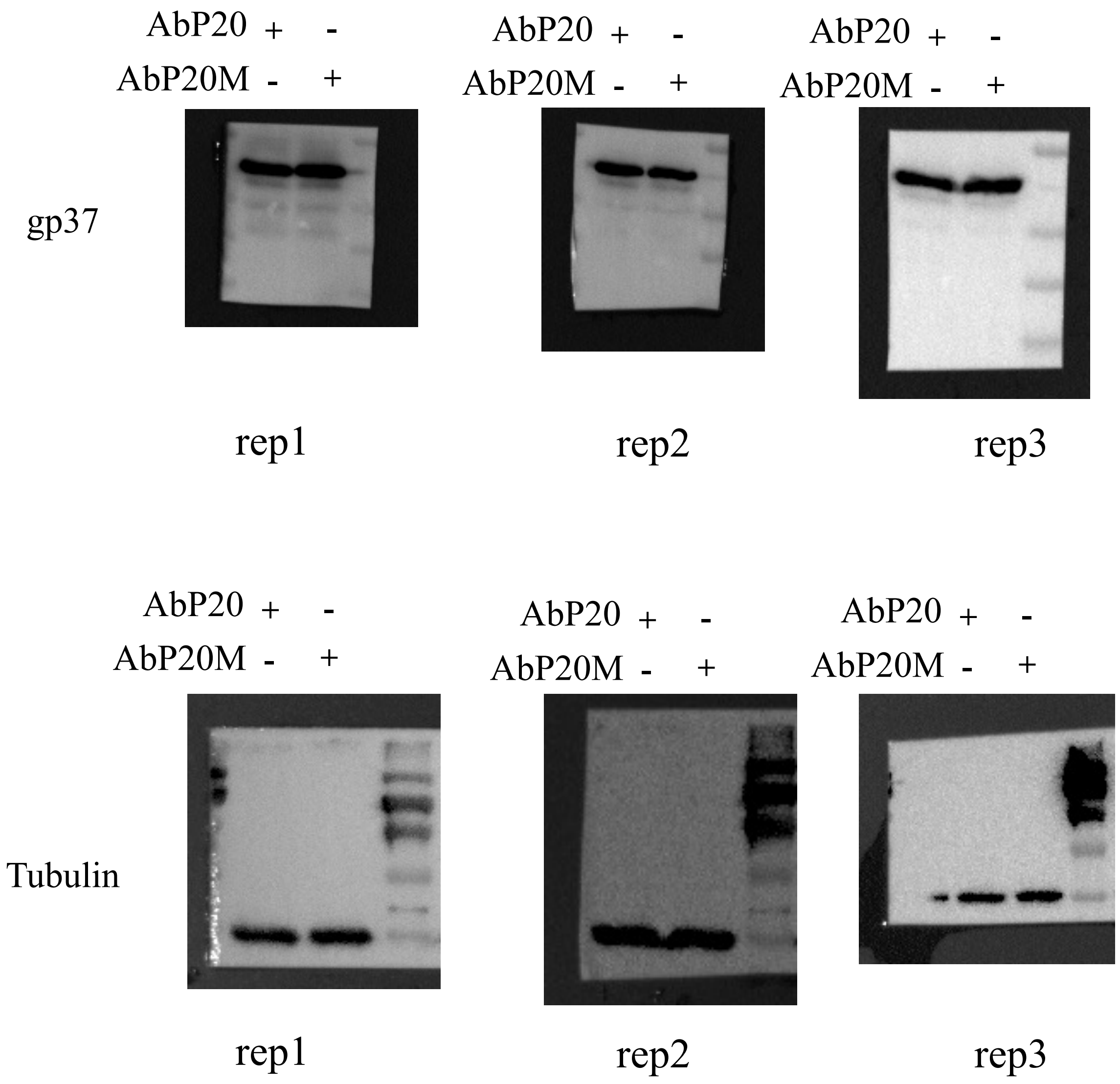

FigS9B Western blot
