# Supplementary material for: Antibodies targeting phage fiber and nozzle proteins impair Acinetobacter baumannii phage therapy by blocking infection and promoting immune clearance
Source: PLoS Biol. 2026 Sep 22;24(9):e3004009. doi: 10.1371/journal.pbio.3004009 (PMC13623143; doi:10.1371/journal.pbio.3004009)
Supplement: Supplementary file 2 [file pbio.3004009.s010.docx]

**S1_Table. Primers used in this study.**

| Primer | Forward sequence (5’-3’) | Reverse sequence (5’-3’) |
| --- | --- | --- |
| 28a-gp35 | AACTTTAAGAAGGAGATATACCATGGATATGAAGTCCAAAGGAAATGAT | TGGTGGTGGTGGTGCTCGAGTTGAATACCTGCTGCCAATT |
| 28a-gp37 | AACTTTAAGAAGGAGATATACCATGGATATGGCAGGTGCTAATTACTTC | TGGTGGTGGTGGTGCTCGAGAGGTGTAACTTGTTCAGTAATAC |
| 28a-gp39 | AACTTTAAGAAGGAGATATACCATGGATATGCAACTATTCGCACGTTGC | TGGTGGTGGTGGTGCTCGAGTCGTCTTAAATATCGTTGGAAG |
| 28a-gp40 | AACTTTAAGAAGGAGATATACCATGGATATGATTCTTGAGGGAGTGTAC | TGGTGGTGGTGGTGCTCGAGTAAACGTCTCCGTCCTCTGTT |
| 28a-gp44 | AACTTTAAGAAGGAGATATACCATGGATATGAATATACTACGCTCATTT | TGGTGGTGGTGGTGCTCGAGAGTTGGATATATTTGACCCGCT |
| gp40-det | CAAGAGAATATGCGTAATCGTAGAT | GATAATAGCACCATAGGCTGCAC |
| gp44-det | GGAGAGCCAGACTTAATTACGCT | GAACTGATCTAGCATGTTCATTTAGT |
| AbP20qP | GAGTTCCACGACGGCTTCTTCG | TAGGATGATGTAATAACGCTGAG |
| Ab28rpob | GGTAAACCTGAGTTCGATGTACG | CCGTTGATAACAAAGGTACCAT |
