# Supplementary material for: Antibodies targeting phage fiber and nozzle proteins impair Acinetobacter baumannii phage therapy by blocking infection and promoting immune clearance
Source: PLoS Biol. 2026 Sep 22;24(9):e3004009. doi: 10.1371/journal.pbio.3004009 (PMC13623143; doi:10.1371/journal.pbio.3004009)
Supplement: Supplementary file 3 [file pbio.3004009.s015.docx]

**Response to reviewers**

**Reviewer #1:**

*The manuscript by Xue and colleagues explores how phage-targeting antibodies might attenuate the efficacy of phage therapy using an Acinetobacter-targeting phage and immunization of mice with individual phage proteins. Structure-guided antigen screening identified predicted nozzle and tail fiber proteins as responsible for eliciting antibodies that attenuated the efficacy of phage therapy in mice. The manuscript is clearly presented and the experiments are logical and reasonably well described. Nonetheless, the study would benefit from addressing the following comments:*

**Major comments:**

*1. The experimental system is somewhat artificial, since patients would never be "pre-vaccinated" with phage before their infection. I understand that this is done to study the effects of antibodies against individual phage proteins, but the translational relevance is still somewhat questionable.*

Response: We highly appreciate this insightful and critical comment regarding the translational limitation of our model. We fully agree that healthy patients never receive prophylactic phage immunization prior to bacterial infection in clinical practice, and the pre-immunization mouse model adopted herein is relatively artificial compared to real-world phage therapy scenarios. Notably, this pre-immunization design was adopted for the following two clinical-mimicking purposes, which guarantee its clinical translational value:

First, this model mimics repeat-cycle phage therapy, the most mainstream clinical administration regimen for drug-resistant *Acinetobacter baumannii* infections. Clinically, patients with persistent or recurrent multidrug-resistant bacterial infections always receive multiple rounds of phage infusion. The first cycle of phage administration elicits robust phage structural protein-specific adaptive antibodies *in vivo*, and these pre-existing antibodies may profoundly compromise the efficacy of subsequent phage treatment. Our pre-immunization model precisely simulates this clinically common secondary-treatment scenario.

Second, this model also simulates patients with prior phage-specific antibody exposure from dual sources. Apart from spontaneous contact with free lytic *A. baumannii* phages distributed in hospital wastewater and ward environments, evidence has confirmed that antibodies targeting bacterial endogenous prophages also contribute to clinical phage therapy failure (*Gordillo Altamirano F, Subedi D, Beiers M, Bucher M, Dahlman S, Patel DM, et al. Cross-reactive anti-prophage antibodies and bacterial heteroresistance implicated in phage therapeutic failure. Nat Med. 2026*). Most clinical *A. baumannii* isolates carry abundant integrated prophage fragments within their genomes. Colonized or infected patients can generate cross-reactive neutralizing antibodies against these intrinsic prophage proteins during bacterial infection, prior to receiving exogenous therapeutic phage infusion. Taken together, both free environmental phages and bacterially carried prophages can trigger pre-existing phage-targeted antibodies, which perfectly matches the antibody-positive state simulated by our pre-immunization model.

Collectively, although the model established in our study cannot mimic first-time phage treatment in antibody-naive patients, it possesses reliable translational significance for repeated phage therapy and prior antibody-exposed patient cohorts. We have clarified this limitation explicitly in the Discussion section (Lines 512-519) in the revised manuscript.

*2. The negative staining shown and the resulting aggregate structures proposed is highly spectulative. Conclusions regarding the orientation of phages in aggregates should not be made based only on the TEM data shown in Figure 4. Either the claims need to be softened or additional experiments to show the proposed structures are needed.*

Response: Thank you for this valuable comment. We agree that the original electron micrographs lacked sufficient clarity. Accordingly, we repeated negative-stain electron microscopy using purified antibodies, and the updated images display clearer structural characteristics of phage aggregation in the presence of varied structural protein-elicited antibodies in revised Fig. 4B and Fig. S8 (see below figure).

**Fig. S8. Different IgG antibodies induce aggregation of phages.** Representative images of AbP20 aggregates in the presence of varied IgGs. Model manners of phage conjugation under each condition are presented above each image. Scalar bar: 100 nm.

*3. The assumption is made throughout the manuscript that observed effects from serum of immunized animals is due to the phage- or phage protein-specific antibodies in the serum. These claims would be stronger if antibodies were purified from the serum and tested on their own.*

Response: Thank you for this important suggestion. Following your suggestions, we further purified specific antibodies from immune serum via Protein G affinity resin and conducted subsequent functional validation. Consistent phenotypic results were observed, including intact antibody-mediated neutralizing activity, enhanced phage aggregation, and facilitated macrophage-mediated phagocytosis (revised Fig. 4B and Fig. 5, Supplementary Fig. S7 and Fig. S8). The detailed experimental methods are also presented accordingly in the revised manuscript.

**Minor comments:**

*1. Line 115-116 - Please include a basic description of the mouse model and state the number of mice per group in the text; this information is in the Figure 1 legend but it should appear in the text as well.*

Response: Revised as suggested all through the revised manuscript.

*2. The fiber and nozzle proteins are sometimes called as such and sometimes called gp40 and gp44, which is confusing and makes it difficult to follow the text at times.*

Response: Thank you for this valuable comment on nomenclature standardization. We completely agree that the mixed use of protein common names and gene locus tags causes reading confusion throughout the manuscript. To unify writing specifications and improve readability, we have revised the full-text naming rule uniformly: we adopt the unified form nozzle protein (gp40) and fiber protein (gp44) for the first appearance in each section and uniformly use gp40/gp44 for subsequent mentions in the main text, figures and figure legends. All inconsistent protein labels have been fully corrected in the revised manuscript.

*3. The reason for selecting the specific proteins used here for immunization should be described, rather than saying they were chosen based on "previous analysis."*

Response: Thank you for the constructive comment. We apologize for the vague description regarding protein selection criteria. We have supplemented and clarified the explicit selection basis in the revised manuscript (lines 183-185).

*4. Figure 2D-F - These plots appear to have identical values between replicates for many of the samples. This is not expected from ELISA data and raw data are not provided so they can't be assessed.*

Response: Thank you for this critical comment. We apologize for the misleading data presentation. Notably, the plotted ELISA data are converted logarithmic values of endpoint antibody titers, instead of raw OD450 absorbance values. Identical titer values among biological replicates are reasonable under identical serum dilution conditions, which caused visual confusion in the original graphs. To improve data transparency, we have supplemented all original raw OD450 scanning data and detailed replicate values below for independent evaluation.

**Raw data for Fig2C（original Fig2E）**

**Raw data for Fig2D（original Fig2F）**

**Raw data for FigS4B（original Fig2D）**

-----------------------------------------

**Reviewer #2:**

*The question asked by the authors is important, as immune response of animal/human organisms to bacteriophages is still poorly understood. The authors analyzed adaptive immune response to selected proteins of the coat of bacteriophage AbP20, infecting Acinetobacter baumannii. Interestingly, antibodies recognizing some proteins were neutralizing, while those recognizing others did not prevent effective infection of the bacterial host. An interesting hypothesis potentially explaining this diversity is proposed. Nevertheless, major revisions are necessary, and important points must be addressed.*

**Major critical points:**

*1. The authors indicated that the AbP20 bacteriophages was characterized in their previous work. However, such a work was not cited. Moreover, I was unable to find any paper describing AbP20 in the literature (using different databases). Therefore, the information is confusing, and it is very unusual to present the whole work based on a subject (bacteriophage in this case) which was not published previously while it is described as being charecterized previously. Therefore, citation of the paper describing characterization of AbP20 is mandatory. If the AbP20 characterization was not published previously, the manuscirpt should be reformatted, and description of the isolation and characterization of this phage must be included.*

Response: Thank you very much for this careful and critical comment. We sincerely apologize for the confusing statement. The preliminary characterization of phage AbP20 has not yet been published in a separate prior study, so our previous wording implying prior published characterization was inappropriate and misleading. Accordingly, we have restructured the manuscript to integrate the full isolation and basic characterization data for AbP20 directly into the main text. We reorganized and supplemented the relevant morphological and genomic characterization results in revised Figure 1, and added a detailed description of phage isolation, purification and preliminary identification in the revised manuscript (lines 122-128) to fully clarify the background of this phage.

*2. The major problem is interpretation of results presented in Figure 4B. From the presented microphotographs, it is not possible to deduce about the pattern of aggregates, and whether AbP20 virions are connected by ther tails or heads. Cam the authors present micrographs with significantly higher resolution? If not, the conclusions based on pictures shown in Figure 4B are just severe overinterpretation of the results.*

Response: Thank you for this critical remark. We fully agree that the original micrographs lacked sufficient resolution to precisely resolve the structural arrangement of phage aggregates and distinguish head–tail contact sites between virions. Following the suggestion raised by another reviewer, we repeated negative-stain transmission electron microscopy with purified specific antibodies and acquired high-resolution micrographs that clearly display the spatial arrangement of aggregated phage particles (revised Fig. 4B; Supplementary Fig. S8). Meanwhile, we have cautiously revised and moderated the relevant descriptive conclusions to avoid overinterpretation in the main text (Lines 308-315) and now it is read as:

Detailed observation of antibody-mediated phage aggregates indicated that gp37-elicited antibodies triggered phage assembly into structures featuring head-to-head juxtaposition, with the spike-like phage tails exposed outward. In contrast, antibodies targeting gp40 and gp44 mediated tail-to-tail phage clustering, where phage spherical capsids were oriented toward the exterior (Fig. S8).

**Fig. S8. Different IgG antibodies induce aggregation of phages.** Representative images of AbP20 aggregates in the presence of varied IgGs. Model manners of phage conjugation under each condition are presented above each image. Scalar bar: 100 nm.

**Minor critical comments:**

*3. The title should include names of the bacteriophage and its bacterial host.*

Response: Following your suggestions, the title has been changed as “Fiber- and nozzle-induced neutralizing antibodies against an *Acinetobacter baumannii* phage impair phage therapy by inhibiting phage proliferation and promoting immune clearance”.

*4. In experiements with mice, the time course is crucial, and should be mentioned in the Abstract.*

Response: Thank you for your valuable suggestion. We have supplemented key immunization time-course information for the mouse experiments within the revised Abstract (lines 35-36).

*5. Line 72 - it would be more appopriate to indicte 80% of patiernts instead of "four patients" or number of patients, instead of percentage, should be indicated in line 70.*

Response: Revised as suggested in the revised manuscript (lines 72-74).

*6. Line 77 - "a Pseudomonas phage"; it is important to indicate what specific phage are you talking about.*

Response: Thank you for your suggestion. The detailed information of the phage mentioned, i.e., PB1-like pseudomonas phage, has been added to the revised manuscript (line 80).

*7. Line 80 - the same as in p. 6, but considering "a staphylococcal phage".*

Response: Revised as “the staphylococcal phage A3R” in the revised manuscript (line 83).

*8. Line 81 - please indicate what specific protein of T4 is mentioned.*

Response: Has been clarified as “the head proteins gp23 and Hoc of T4 phage” in the revised manuscript (line 85).

*9. Line 89 - Please, be more specific (in fact, this concerns the whole manuscript); what do you mean by "specific immue responses"? In fact, it should be briefly described what pathways of the innate immune response are activated by bacteriophages. Moreover, it should be indicated whether these pathways are complete and lead to massive production of cytokines or thy are somehow silenced.*

Response: Thank you for this constructive comment. We acknowledge that the original phrase “specific immune responses” was overly vague. We have supplemented detailed clarifications on Lines 88–102 in the revised manuscript, explicitly defining phage protein-driven adaptive antigen-specific immunity, summarizing the major innate immune signaling cascades triggered by phage uptake, and clarifying the activation status of these immune axes. The revised text elaborates on phage-initiated innate immune sensing, downstream inflammatory cytokine secretion profiles, intact signal transduction cascades, and subsequent antigen presentation that bridges innate activation to phage-specific adaptive antibody responses, distinguishing robust cytokine induction from immune-suppressed signaling states.

*10. Line 94 - Did you mean "the adaptive immune response" rather then "the immune response"?*

Response: Correct. This is also corrected in the revised manuscript (line 99).

*11. Line 112 - "AbP20-inducted", instead of "AbP20 induced" (this changes the meaning of the title).*

Response: Thank you for this careful proofreading. We apologize for this grammatical error caused by carelessness. We have revised the incorrect expression to the proper compound adjective form AbP20-induced in the revised manuscript (lines 121 and 158).

*12. Line 165 - Add "genes coding for" after "expressed". Please, remember that genes are expressed, not proteins. Proteins, as products of gene expression, can be produced or synthesized, but not expressed (in other words, only genes are expressed, not proteins; "protein expression" is a jargon which should be avoided).*

Response: Thank you for this rigorous academic correction. We fully agree with this precise terminology standard and apologize for the inaccurate wording regarding molecular expression logic. We have revised the sentence strictly following your suggestion by inserting “genes coding for” immediately after “expressed” in the revised manuscript (line 186).

*13. Figure 5 presentes an interesting hypothesis, but the results presented in this paper cannot corroborate it. The presented analysis of micrographs from Figure 4B is an overinterpretation of the results. More convincing micrographs (with significantly higher resolution) should be demonstrated to show testing of this hypothesis.*

Response: Thank you for this critical comment. We fully recognize that the original low-resolution micrographs were insufficient to robustly validate the proposed mechanistic hypothesis and led to overstated morphological interpretation. Address to this, we repeated negative-stain electron microscopy with purified antibodies and acquired high-resolution imaging data that better resolve the spatial arrangement of antibody-mediated phage aggregates (revised Fig. 4B; Supplementary Fig. S8). Corresponding textual descriptions have been moderated and refined to adopt more cautious phrasing at Lines 308-315, to avoid definitive causal conclusions beyond observable structural features.

Furthermore, we have toned down the schematic illustration and interpretive statements for the original Figure 5 (renumbered Figure 7) accordingly at Lines 424-433 and now it is read as:

Based on all these observations, we propose a working mechanistic scenario for antibody-mediated phage therapy outcomes. Antibodies targeting distinct structural proteins of phage AbP20 exert disparate regulatory effects on phage fate and therapeutic performance. Specifically, antibodies against the capsid protein (gp37), portal protein (gp35) and adaptor protein (gp39) mainly serve as binding antibodies, which exert negligible impacts on phage infectivity and macrophage-mediated phagocytosis, and barely alter final phage therapeutic efficacy. By comparison, antibodies induced by the fiber protein (gp44) and nozzle protein (gp40) act as dominant neutralizing antibodies. These two types of antibodies suppress phage proliferation via blocking phage-host interaction and accelerate systemic phage clearance by triggering the formation of large phage aggregates, thereby compromise the therapeutic potency of phage AbP20.

*14. Lines 351-353 - see p. 2 and 13.*

Response: The sentence has been revised as the following in the revised manuscript (lines 482-490):

The second mechanism involves antibody-triggered phage aggregation, which alters the physicochemical properties of phage particles and may consequently affect macrophage phagocytic uptake efficiency. This speculative mechanism is partially supported by transmission electron microscopic observations: antibodies targeting fiber and nozzle proteins promoted the formation of large phage aggregates, in which multiple phage particles appeared to cluster with spike-like tails juxtaposed toward one another while spherical capsids faced the aggregate exterior, a spatial arrangement that may facilitate phagocytic clearance. In contrast, relatively smaller phage clusters were detected when phages were incubated with gp37-induced antibodies.

*15. Description of method should be more precise. Current descriptions lack important details.*

Response: Thank you for this valuable reminder. We have thoroughly reviewed the entire Methods section and supplemented critical experimental details to improve methodological rigor and reproducibility.

*16. Line 379 - Where was AbP20 published? See p.1.*

Response: Thank you for your careful review. Consistent with the revision addressed in Major Critical Point 1, we have removed the statement implying prior independent publication of AbP20 characterization data, as this phage has not been formally characterized in a separate published work. We have integrated complete isolation, purification and preliminary characterization data for AbP20 into the main text with restructured characterization results presented in revised Figure 1, and detailed experimental background is fully supplemented on Lines 122-128 to fully document the origin and basic traits of this phage within the current manuscript.

*17. Line 379 - Replace "logarithmic culture" with "Exponentially growing culture".*

Response: Revised as suggested all through the revised manuscript.

*18. Line 385 - Provide conditions for ultracentrifugation.*

Response: Thank you for this precise suggestion. We have supplemented detailed ultracentrifugation parameters including rotational speed and duration in the revised manuscript (line 538).

*19. Line 405 - Again, genes are expressed, not proteins (see p. 12).*

Response: Thank you for pointing this out. We have revised the corresponding description in the revised manuscript (line 562).

*20. Line 406 - Proteins cannot be cloned! The indicated vector is a plasmid, being a DNA molecule, and no protein can be cloned into DNA. Obviously, genes coding for these proteins could be cloned.*

Response: Thank you for this precise academic correction. We sincerely apologize for this inaccurate wording that confuses gene cloning and protein expression. We have revised the sentence strictly to state that the genes encoding the target structural proteins were cloned into the designated plasmid vector in the revised manuscript (lines 562-564).

*21. Supplementary material contains some results showing characterization of AbP20. However, they are just raw results, without any description in the text. This is confusing. Was charactyerization of this phage previously published? If not, building a hypothesis on the basis of weakly characterized model is questionable.*

Response: Thank you for this thoughtful comment. We apologize for the confusion caused by unannotated raw characterization data placed only in supplementary materials without textual explanation. As addressed in Major Critical Point 1 and Comment 16, phage AbP20 has not been published in any prior independent study. To resolve insufficient characterization concerns and integrate scattered data, we have consolidated all key morphological, genomic and biological characterization data from the supplement into the main text, restructured and unified these results in revised Figure 1, and supplemented comprehensive textual descriptions covering phage isolation, morphology and genomic features in the revised manuscript (Lines 122-128). With complete and formally documented basic characterization within the present manuscript, the reliability of our subsequent functional and mechanistic hypotheses is fully supported.

-----------------------------------------

**Reviewer #3:**

*This is an interesting and potentially impactful study examining how antibodies directed against specific phage structural proteins influence therapeutic efficacy. The single-phage focus is appropriate, and the manuscript provides convincing evidence that antibodies against phage, specifically its gp40 and gp44, are associated with reduced phage activity, increased macrophage uptake, reduced splenic titers, and impaired therapeutic outcomes. The core observation can differentially affect phage therapy outcomes and represents a meaningful contribution to the field.*

*However, the manuscript does not yet reach the level of mechanistic resolution expected. While multiple phenomena are documented, most are causal relationships between neutralization, aggregation, and immune clearance, and several mechanistic interpretations are therefore stronger than the data directly support. Most conclusions rely on correlated observations across assays rather than direct causal tests. Addressing this, either experimentally and/or through more cautious interpretation with literature support, would substantially strengthen the study.*

**Major comments**

*1. Mechanistic relationships between neutralization and clearance are not defined. The central conclusion is that gp40- and gp44-specific antibodies impair therapy through both neutralization and enhanced clearance. While each effect is supported individually, their relative contributions are not established. Whether therapeutic impairment is driven primarily by reduced infectivity, increased clearance, or a combination of both are important determinations, and whether these processes are linked or independent. A clearer dissection is needed.*

Response: Thank you for this pivotal comment regarding the relative contributions and mechanistic linkage between antibody-mediated phage neutralization and accelerated immune clearance. To disentangle these two effects and quantify their individual and combined impacts on therapeutic failure, we constructed an antibody escape mutant phage (AbP20M) via iterative adaptive mutagenesis under escalating antibody selective pressure (revised Fig. 6A, see below figure). Sequencing confirmed point mutations within gp44 fiber protein (Q131K) and gp40 nozzle protein (Y305H, T656G) (revised Fig. 6B, see below figure), and functional assays verified that AbP20M achieved a several-hundred-fold reduction in susceptibility to antibody neutralization (revised Fig. 6C, see below figure).

Notably, the mutant phage still underwent antibody-triggered aggregation and macrophage phagocytosis to a comparable degree as wild-type AbP20, with equivalent *in vivo* clearance rates observed between AbP20 and AbP20M (revised Fig. 6D, Fig. S12). In antibody-pretreated therapeutic mouse models: at a low MOI of 10, only one mouse survived in the AbP20M group (1/6), likely due to saturation by high antibody titers that overcame neutralization escape capacity (revised Fig. 6H, see below figure). At a higher MOI of 100, however, 50% survival was observed in AbP20M-treated group (revised Fig. 6I, see below figure).

Collectively, these loss-of-neutralization experiments with fixed clearance kinetics demonstrate that attenuating antibody neutralization only partially rescues therapeutic efficacy. The two processes are mechanistically interconnected rather than independent, and their synergistic effect leads to the maximum attenuation of phage therapeutic performance. We have integrated their interdependency into the main text for explicit clarification (lines 366-402).

**Figure 6. AbP20M with improved neutralization escape ability ameliorates antibody-mediated therapeutic damage.** (A) Schematic diagram showing the in vitro evolution of AbP20. AbP20‑immunized serum was serially diluted from 1:800 to 1:100 (with triplicate screening at 1:100), incubated with phage AbP20, and allowed to infect A. baumannii Ab28; 10 plaques were picked per round for serial passage under increasing selection pressure for EOP and sequencing analysis. (B) The mutation sites of AbP20M. (C) AbP20M shows significantly enhanced antibody-induced neutralization escape efficiency. (D) Representative images of AbP20M and AbP20 aggregates in the presence of varied IgGs. Scalar bar: 100 nm. (E) Size distribution of AbP20M aggregates in the presence of varied IgGs. The intensity distributions of the PBS‑treated group and AbP20 aggregates are indicated by dashed lines in different colors. (F-H) Effect of varied IgGs on the phagocytosis of AbP20M and AbP20 by RAW264.7 cells. Intracellular phages were detected using rabbit anti‑gp37 antibody, with Tubulin serving as a reference. Band intensities of gp37 from three independent Western blots were analyzed by Image J software and compared. (I) Schematic diagram presenting the evaluation of AbP20M in mouse model. Mice received two rounds of intramuscular immunization. Serum was collected 3 days prior to bacterial challenge for antibody titer detection. Subsequent treatment and sampling procedures were consistent with the previous protocol (n = 6). (J) Anti-phage IgG antibodies detected by ELISA, with an initial dilution factor of 10. (K) Neutralization effects of AbP20-immune serum detected by EOP assay. (L) Survival rates of mice in a bacteremia model. Mice were immunized with wild‑type AbP20 and subsequent treatment with varied dose of AbP20 or AbP20M. (M) Bacterial burden in the liver, spleen, and kidneys from mice receiving 100 MOI AbP20M treatment (n = 4). Unpaired Student’s t tests. ns: not significant, *p < 0.05, and ****p < 0.0001.

*2. Antibody-dependent aggregation, increased macrophage-associated phage signal, and reduced in vivo titers are all shown. However, it is not demonstrated that aggregation itself drives uptake. The current data do not distinguish between effects of aggregate structure and Fc-mediated opsonization. This distinction is important for the proposed model.*

Response: Thank you for this insightful comment. We fully agree that separating the contribution of phage structural aggregation from Fc-dependent opsonization is essential to validate our mechanistic model. To resolve this ambiguity, we designed two complementary sets of experiments to dissect these two pathways independently.

First, we conducted Fc receptor blocking assays in macrophage cultures. Following effective Fc receptor blockade, phage internalization and systemic clearance rates showed no significant difference relative to the untreated control group, indicating that Fc-mediated opsonization is not the dominant pathway driving enhanced phage uptake (revised Fig. 5A, see below figure). To further confirm this conclusion and eliminate concerns about incomplete receptor blockade, we digested intact purified IgG with papain to generate Fab fragments lacking the Fc domain (revised Fig. 5B, see below figure). Functional validation confirmed that Fab fragments retained capacity to neutralize AbP20 infectivity (revised Fig. 5C, see below figure). Nevertheless, Fab treatment failed to induce robust phage aggregation and could not facilitate macrophage phagocytosis of phage particles (revised Fig. 5D-H, see below figure).

Combined, these data confirm that phage clustering triggered by bivalent full-length antibody crosslinking, rather than Fc-dependent opsonization, is the key factor promoting macrophage uptake. We have integrated this key mechanistic distinction and supporting experimental results into the main text (lines 326-364) to strengthen the reliability of our proposed working model.

**Figure 5.** **Size of antibody-induced phage agglutination regulates phagocytosis in an Fc receptor-independent manner.** (**A**) Effect of immune serum on the phagocytosis of AbP20 by macrophages in the presence of Fc receptor blocker. Intracellular phages after incubation with Fc receptor blocker (Anti‑mouse CD16/CD32) or its isotype were detected using rabbit anti‑gp37 antibody, with Tubulin serving as a reference. Band intensities of gp37 from three independent Western blots were analyzed by Image J software and compared with those of the PBS‑treated control group. (**B**) Preparation of IgG Fab fragments. Papain and purified IgGs were mixed at a ratio of 1:10 and incubated at 37 °C for 12 hours. After purification, Fab fragments were obtained and verified by SDS‑PAGE analysis. (**C**) Effects of varied Fabs on the efficiency of plating (EOP) of phage AbP20. (**D**) Diameter distribution of AbP20 aggregates in the presence of different Fabs or IgGs. The intensity distribution of the PBS‑treated group is shown as a dashed line, serving as a reference for the other groups. (**E-H**) Effect of Fabs or IgGs on AbP20 phagocytosis by RAW264.7 cells. Intracellular phages were detected using rabbit anti‑gp37 antibody, with Tubulin serving as a reference. Band intensities of gp37 from three independent Western blots were analyzed by Image J software and compared with those of the PBS‑treated control group. Unpaired Student’s t tests. ns: not significant, *p < 0.5, **p < 0.01, and ****p < 0.0001.

*3. In vivo clearance mechanism is inferred rather than directly tested. Reduced splenic phage titers following immunization are consistent with enhanced clearance, but the mechanism is not directly tested in vivo. It is therefore not clear whether phagocytosis, aggregation, or other antibody-dependent processes are responsible.*

Response: Thank you for this critical comment. We fully agree that original splenic viable phage titer data only provided indirect evidence for in vivo phage clearance and failed to dissect the underlying antibody-dependent clearance. To address this gap, we dynamically detected viable phage abundance in mouse spleen across sequential time points. Consistently decreased splenic infectious phage titers were observed in immunized groups, which preliminarily verified accelerated phage elimination after antibody induction (revised Fig. 4I).

Combined with our in vitro Fc-blocking and Fab fragment experiments, we deduce that antibody-induced phage aggregation, instead of Fc-mediated opsonization, serves as the dominant driver for in vivo splenic phage clearance.

*4. The reduction in EOP is interpreted as inhibition of phage-bacteria interaction, but the affected step is not identified. Neutralization could occur at adsorption, DNA injection, or another stage of infection. Without direct assays, this remains unclear.*

Response: Thank you for this valuable comment. We agree that original EOP reduction data could not pinpoint the exact infection stage targeted by antibody-mediated neutralization. To resolve this ambiguity, we performed staged infection assays to separately assess the impacts of structure-specific sera on phage adsorption, genomic DNA injection, and intracellular progeny phage release throughout the lytic infection cycle.

Consistent with sera against intact AbP20 virions, sera specific for the fiber protein (gp44) significantly impeded phage adhesion to host bacterial cells (revised Fig. 2H, see below figure), which further blocked subsequent phage genomic DNA injection into bacteria (revised Fig. 2I, see below figure). Differentially, the nozzle protein (gp40)-targeted sera exerted no obvious influence on phage host adsorption, but specifically suppressed downstream DNA injection processes (revised Fig. 2I, see below figure). Both adsorption inhibition and post-adsorption injection inhibition ultimately contributed to suppressed progeny phage production and release (revised Fig. 2J, see below figure).

These stage-specific assay results align well with the structural localization and biological functions of gp40 and gp44 on the AbP20 virion. Collectively, these data verify that structure-specific antibodies disrupt discrete steps of phage-host interactive processes to mediate phage neutralization, which accounts for the decreased EOP phenotype observed in our study.

**Figure 2. Fiber- and nozzle-induced antibodies lead to the failure of phage therapy.** (H) Effect of serum against different components of AbP20 on phage adsorption. Serum diluted 100‑fold were incubated with 10⁸ PFU of AbP20 at 37 °C for 1 h, followed by incubation with 10⁸ CFU/mL of Ab28 at 4 °C for 2 h. After washing with PBS, the samples were analyzed by Western blotting. Gray values were calculated using ImageJ software and statistically analyzed by comparing to the PBS-treated group. (I) Effect of serum against different components of AbP20 on phage DNA injection. Sera diluted 100‑fold were incubated with 10⁸ PFU of AbP20 at 37 °C for 1 h, followed by incubation with 10⁸ CFU/mL of Ab28 at 37 °C for 20 min. After washing with PBS, the copies were determined by qPCR, and the inhibition rate was calculated and statistically analyzed by comparing to the PBS-treated group. (J) Effect of serum against different components of AbP20 on phage release. Serum diluted 100‑fold were incubated with 10⁸ PFU of AbP20 at 37 °C for 1 h, followed by incubation with 10⁸ CFU/mL of Ab28 at 37 °C for 2 h. After centrifugation, phage titers in the supernatant were determined using the double‑layer agar plate method and statistically analyzed by comparing to the PBS-treated group. Unpaired Student’s t tests. ns: not significant, *p < 0.05, **p < 0.01, ***p < 0.001, and ****p < 0.0001.

*5. The manuscript presents multiple related observations (neutralization, aggregation, uptake, and clearance) but these are not fully integrated into a coherent mechanistic framework that would relate to therapeutic settings. The Discussion would benefit from a clearer model and more explicit framing of remaining questions.*

Response: Thank you for this insightful comment. We fully acknowledge that original manuscript scattered independent phenotypic results without assembling a unified, therapy-oriented mechanistic axis, and failed to clarify unresolved research limitations. To address this shortcoming, we have built an integrated working mechanistic framework and clarified pending research questions in the revised Discussion section (Lines 499-510), linking all core observations to practical phage therapy scenarios as shown below:

In brief, phage structural protein-specific antibodies impair phage therapy via two synergistic, interconnected pathways: targeted infectivity neutralization and aggregation-dependent immune clearance. First, anti-gp44 fiber antibodies block phage adsorption onto host bacteria, while anti-gp40 nozzle antibodies inhibit phage DNA injection post bacterial binding; both patterns achieve phage neutralization and lowered EOP. Second, bivalent full-length antibodies trigger distinct phage aggregation patterns based on targeted protein epitopes. Head-to-head phage aggregation induced by gp37-targeted antibodies exerts minor therapeutic impairment, as this aggregation pattern barely hinders phage-bacterium binding and does not facilitate macrophage phagocytic uptake. By contrast, tail-to-tail phage clustering mediated by gp40 and gp44 antibodies aggravates therapeutic failure dual-modally: aggregated phage particles lose efficient bacterial adsorption capacity to amplify neutralization efficacy, and enlarged phage clusters are preferentially engulfed and cleared by macrophages independent of Fc-mediated opsonization.

Moreover, we have explicitly summarized key unresolved questions and future research directions in the revised Discussion, including phage morphological variability, in vivo dynamic balance between phage mutation escape and antibody pressure, and translational strategies to circumvent antibody-mediated phage elimination for optimized phage therapy.

*6. The dominance of gp40- and gp44-specific effects is likely influenced by the structural organization of this phage. The broader implications across different phages are only briefly mentioned and should be expanded.*

Response: Thank you for this valuable suggestion. We fully agree that the prominent neutralizing and aggregative effects triggered by gp40 nozzle and gp44 fiber antibodies are highly dependent on the unique structural topology of podovirus AbP20, and the cross-phage applicability of our findings was insufficiently elaborated in the original manuscript. To enrich the translational breadth of this work, we have expanded discussions regarding phage-conserved structural features and generalized research implications in the revised Discussion section (Lines 494-498).

Specifically, nozzle and distal fiber proteins are functionally conserved across most Gram-negative targeting phages, which govern host recognition, anchoring and genome ejection universally. This structural conservation indicates that nozzle/fiber-specific adaptive antibodies may serve as a common barrier compromising podophage-based phage therapy. Nevertheless, the antibody-induced aggregation pattern is strain-specific: the tail-associated epitope distribution of AbP20 facilitates tail-to-tail phage crosslinking upon antibody binding, while siphophages or myophages with distinct tail architectures tend to form disparate aggregate morphologies, which may lead to divergent phagocytosis efficiency and therapeutic outcomes. Besides, variable surface epitope accessibility among diverse phage isolates also alters the dominant immunodominant protein types, thereby reshaping antibody-mediated interference patterns.

**Additional comments**

*7. No endotoxin quantification or toxin controls are provided. Given the reliance on macrophage and immune readouts, this should be clarified or acknowledged as a limitation.*

Response: Thank you for this important reminder. We acknowledge that the original manuscript lacked endotoxin detection data and toxin control groups, which is a critical omission for macrophage-based immune phenotyping assays, as residual endotoxin may interfere with innate immune activation and phagocytic phenotypes. To rule out endotoxin-mediated immune interference, we quantified residual endotoxin levels in ultracentrifugation-purified AbP20 phage stock. Endotoxin quantification results showed that the purified phage stock contained 762.32 EU/mL endotoxin at a phage titer of 2 × 10^11^ PFU/mL, while the working phage concentration adopted for all cellular and in vivo assays yielded a final endotoxin level of 76.23 EU/mL. Notably, equal dosages of standardized AbP20 phage working solution were administered across all experimental and control groups, ensuring uniform endotoxin exposure among all groups.

We have added these endotoxin quantification data in the main text of the revised manuscript (lines 245-246). Meanwhile, we have acknowledged trace residual endotoxin as a minor inherent limitation of phage purification in the Discussion section and clarified that unified endotoxin dosage eliminates its confounding effect on inter-group immune comparison results (lines 468-471).

*8. The decrease in neutrophil percentage alongside increased total WBC suggests a shift in leukocyte composition rather than normalization. This pattern is not explained, and its restriction to the MOI 10 group is unclear. Absolute counts would improve interpretation. There also appears to be an inconsistency in reported timing (10 h vs 24 h) that should be resolved.*

Response: Thank you for this careful and critical observation. We sincerely apologize for the confusing leukocyte data presentation, and inconsistent sampling time points described in the original manuscript. To eliminate misinterpretation derived from relative cellular proportions, we have re-analyzed and reorganized all peripheral blood immune cell data by adopting absolute leukocyte counts instead of percentage values, and rearranged corresponding illustrative figures accordingly (revised Fig. 1E-G, Fig. S2).

Revised absolute count data clarified that peripheral neutrophil absolute quantities stayed within physiological healthy ranges across all experimental groups with no significant aberrant alteration. The reduced neutrophil percentage exclusively observed in the MOI 10 group was merely a byproduct of elevated total WBC abundance, rather than genuine neutrophil depletion or immune status normalization. The relatively higher WBC and lymphocytes count observed in high-dose phage treatment group (MOI=10) are comparable to the uninfected mock group (revised Fig. 1F-G and Fig. S2), indicating that AbP20 phage at MOI=10 effectively promotes bacterial clearance and protects mice from lethal *A. baumannii* infection.

Furthermore, we have unified the whole-blood sampling timepoint to 10 h post treatment throughout the main text, figure panels and figure legends. The mismatched 24 h labeling in original figure legends was a typographical error, which has been fully corrected in the revised manuscript.

*9. The study relies largely on endpoint measurements. Time-resolved data would help establish the sequence of infectivity loss, aggregation, uptake, and clearance.*

Response: Thank you for this constructive comment. We fully acknowledge that original experiments mostly adopted single endpoint detection, which failed to define the chronological cascade of antibody-triggered phage inactivation, aggregation, cellular uptake and systemic clearance. To map the sequential order of these core events, we supplemented multi-timepoint dynamic assays *in vivo*, which are partially consistent with the time-series sampling design adopted for Comment 3.

We dynamically detected viable infectious phage titers in mouse spleen at 1 h, 3 h, 6 h and 12 h to trace total phage particle fate (revised Fig. 4I). Collectively, these temporal datasets help delineate the sequential cascade: antibody binding induces rapid phage infectivity loss and structural aggregation, followed by accelerated macrophage uptake, and eventual systemic in vivo clearance.

*10. Only total IgG responses are measured. Lack of subclass or Fc-function data limits interpretation of uptake and clearance mechanisms and should be acknowledged.*

Response: Thank you for this critical comment. We acknowledge that original manuscript only quantified total IgG abundance, without profiling IgG subclass distribution or validating Fc effector functions, which restricted mechanistic interpretation of antibody-dependent phage uptake and systemic clearance. To fill this research gap, we have supplemented relevant immunological and functional validation data and clarified related mechanistic discussions in the revised text.

First, we quantified the abundance of major murine IgG subclasses triggered by immunization with whole AbP20 virion and individual structural proteins (revised Fig. S4C-F). Second, we have elaborated the potential linkage between IgG subclass features, antibody structural crosslinking and phage clearance patterns in the revised Discussion section (Lines 481-482). Most importantly, consistent with the supplementary assays addressed in Comment 2, we have completed orthogonal Fc functional verification experiments including Fc receptor blockade and papain-digested Fab fragment assays (revised Fig. 5A-B), which rule out Fc-mediated opsonization as the core driver for macrophage phagocytosis.

Finally, we have explicitly acknowledged that further in-depth Fc receptor affinity assays and subclass-specific immunization tests remain warranted for finer mechanistic dissection and noted this as a clear limitation of the current study in the revised Discussion section (lines 492-494).

*11. The figures are generally readable, but several multi-panel layouts are visually dense and would benefit from a stronger visual hierarchy or splitting.*

Response: Thank you for this thoughtful suggestion on graphical optimization. We appreciate your feedback regarding crowded multi-panel layouts and ambiguous visual hierarchy in partial figures, which may hinder result reading and information acquisition. We have systematically reviewed all main and supplementary figures throughout the manuscript. Specifically, we have redesigned panel arrangement, adjusted element spacing and optimized visual hierarchy to simplify the layout of revised figures, effectively reducing panel crowding and improving graphical readability. And we also minimized graphical clutter via adjusting label font size, color marking and partition lines for these retained layouts to improve visual clarity as much as possible.
