# Supplementary material for: Antibodies targeting phage fiber and nozzle proteins impair Acinetobacter baumannii phage therapy by blocking infection and promoting immune clearance
Source: PLoS Biol. 2026 Sep 22;24(9):e3004009. doi: 10.1371/journal.pbio.3004009 (PMC13623143; doi:10.1371/journal.pbio.3004009)
Supplement: Supplementary file 4 [file pbio.3004009.s016.docx]

**Response to reviewers**

***Reviewer #1:***

*The authors have addressed nearly all of my comments from the initial round of review. Their response to major comment #2 about aggregate structures, however, is not sufficient. Negative stain is highly variable and TEM images of phages often show clustering/aggregation of particles in the absence of IgG. Even with improved images in Fig. 4B, these representative images should not be used to compare aggregate sizes or orientations and attribute differences to phage protein-specific IgG. This issue could be addressed with gold labeling of IgG, or by removing conclusions about phage aggregation and orientation that are only based on the TEM images shown in Fig. 4B.*

**Response:** We greatly appreciate the reviewer’s critical and insightful comments regarding the limitation of TEM images for evaluating phage aggregation and particle orientation. Due to the time issue and the potential technical challenges in determining the orientation of phage aggregates using gold labelled IgG, as suggested by the reviewer, we have substantially revised and softened our relevant conclusions to avoid overinterpretation of the TEM results. Specifically, we clarified that the orientation of phage aggregates induced by varied antibodies is probably existed according to the principle of antibody-antigen binding nature (lines 315-319; line 512), removed the word “head-to-head” from the discussion (line 509), and removed the word “tail-to-tail” from the abstract (line 41) and figure legend for Figure 7 (lines 440-441).

***Reviewer #2:***

*The authors addressed all my previous critical comments. I do not have further concerns, and recommend acceptance of the revised manuscript.*

**Response:** Thank you for all your valuable comments and suggestions.

***Reviewer #3* *(Dwayne Roach)***

*The authors have responded carefully and constructively to my previous comments, and the revised manuscript is substantially strengthened. In particular, I appreciate the considerable additional experimental work undertaken to distinguish the effects of antibody mediated neutralization from enhanced phage clearance, to examine the stage of the infection cycle affected by antibodies targeting different structural proteins, and to investigate the mechanisms underlying antibody enhanced macrophage uptake. Overall, I consider the major concerns from my previous review to have been satisfactorily addressed.*

*I have only a few minor suggestions for the final version. First, the authors should use slightly more cautious language when attributing accelerated in vivo phage clearance, since this mechanism is supported indirectly by the in vitro experiments. Second, the data support a role for antibody mediated aggregation in macrophage uptake, but not necessarily a causal role for specific aggregate geometry. Third, the endotoxin level should be described more neutrally, which now reads as a complete lack of potential cofounder effects. These are minor issues and do not affect the main conclusions.*

**Response:** Thank you for your valuable comments and suggestions, which have helped us to substantially improve our manuscript. According to your comments, we have revised the statements and language in our manuscript to show more cautious perspectives, specifically, we have adopted more cautious and neutral wording when describing the correlation between the antibody-mediated aggregates and macrophage uptake/*in vivo* phage clearance (line 271 and lines 497-498); we have revised the description of endotoxin levels in a more neutral and prudent manner, and objectively elaborated the potential influence of endotoxin on macrophage uptake in the presence of different antisera targeting phage structural proteins, both in the main text (Lines 247–248) and discussion section (Lines 471–474).
