# Supplementary material for: Antibodies targeting phage fiber and nozzle proteins impair Acinetobacter baumannii phage therapy by blocking infection and promoting immune clearance
Source: PLoS Biol. 2026 Sep 22;24(9):e3004009. doi: 10.1371/journal.pbio.3004009 (PMC13623143; doi:10.1371/journal.pbio.3004009)
Supplement: Peer review history

# Editor report: Decision Letter 0

**DOI:** 10.1371/journal.pbio.3004009.r001

**Authors:** Melissa Vazquez Hernandez

12 Feb 2026

Dear Dr Yang,

Thank you for submitting your manuscript entitled "Fiber- and nozzle-induced antibodies impair phage therapy by inhibiting phage proliferation and promoting immune clearance" for consideration as a Research Article by PLOS Biology.

Your manuscript has now been evaluated by the PLOS Biology editorial staff as well as by an academic editor with relevant expertise and I am writing to let you know that we would like to send your submission out for external peer review.

However, before we can send your manuscript to reviewers, we need you to complete your submission by providing the metadata that is required for full assessment. To this end, please login to Editorial Manager where you will find the paper in the 'Submissions Needing Revisions' folder on your homepage. Please click 'Revise Submission' from the Action Links and complete all additional questions in the submission questionnaire.

Once your full submission is complete, your paper will undergo a series of checks in preparation for peer review. After your manuscript has passed the checks it will be sent out for review. To provide the metadata for your submission, please Login to Editorial Manager (https://www.editorialmanager.com/pbiology) within two working days, i.e. by Feb 14 2026 11:59PM.

If your manuscript has been previously peer-reviewed at another journal, PLOS Biology is willing to work with those reviews in order to avoid re-starting the process. Submission of the previous reviews is entirely optional and our ability to use them effectively will depend on the willingness of the previous journal to confirm the content of the reports and share the reviewer identities. Please note that we reserve the right to invite additional reviewers if we consider that additional/independent reviewers are needed, although we aim to avoid this as far as possible. In our experience, working with previous reviews does save time.

If you would like us to consider previous reviewer reports, please edit your cover letter to let us know and include the name of the journal where the work was previously considered and the manuscript ID it was given. In addition, please upload a response to the reviews as a 'Prior Peer Review' file type, which should include the reports in full and a point-by-point reply detailing how you have or plan to address the reviewers' concerns.

During the process of completing your manuscript submission, you will be invited to opt-in to posting your pre-review manuscript as a bioRxiv preprint. Visit http://journals.plos.org/plosbiology/s/preprints for full details. If you consent to posting your current manuscript as a preprint, please upload a single Preprint PDF.

Feel free to email us at plosbiology@plos.org if you have any queries relating to your submission.

Kind regards,

Melissa

Melissa Vazquez Hernandez, Ph.D.

Associate Editor

PLOS Biology

mvazquezhernandez@plos.org

# Peer review: Decision Letter 1

**DOI:** 10.1371/journal.pbio.3004009.r002

**Authors:** Melissa Vazquez Hernandez

1 Apr 2026

Dear Dr Yang,

Thank you for your patience while your manuscript "Fiber- and nozzle-induced antibodies impair phage therapy by inhibiting phage proliferation and promoting immune clearance" was peer-reviewed at PLOS Biology. Your manuscript has been evaluated by the PLOS Biology editors, an Academic Editor with relevant expertise, and by three independent reviewers.

In light of the reviews, which you will find at the end of this email, we would like to invite you to revise the work to thoroughly address the reviewers’ reports. As you will see below, all reviewers recognize the relevance and potential impact of the study, particularly the identification of phage structural proteins that elicit neutralizing antibodies and impair therapeutic efficacy. However, several important concerns need to be addressed to strengthen the manuscript. In particular, Reviewer #3 raises key mechanistic issues that we consider essential, including the need to disentangle the relative contributions of neutralization and immune clearance to therapeutic failure, to clarify whether antibody-dependent aggregation directly drives macrophage uptake versus Fc-mediated opsonization, and to more directly test the in vivo mechanisms underlying phage clearance. In addition, Reviewer #1 and Reviewer #2 both highlight concerns regarding the interpretation of the TEM data, noting that the conclusions about aggregate structure and orientation are currently not sufficiently supported and should either be reinforced experimentally or more cautiously presented. Reviewer #1 also requests validation of antibody-specific effects using purified antibodies rather than serum, while Reviewer #2 raises a critical issue regarding the lack of clear prior characterization or citation of the AbP20 phage, which must be addressed. Across the reports, there are also requests for improved methodological detail, clarification of experimental design and data presentation, and a more integrated and cautious discussion of the findings and their generalizability. We agree with these concerns and consider that addressing them will substantially strengthen the work.

In addition to these revisions, you will need to complete some formatting changes, which you will receive in a follow up email. A member of our team will be in touch with a set of requests shortly.

We appreciate that these requests represent a great deal of extra work, and we are willing to relax our standard revision time to allow you 6 months to revise your study. Please email us (plosbiology@plos.org) if you have any questions or concerns, or envision needing a (short) extension.

At this stage, your manuscript remains formally under active consideration at our journal; please notify us by email if you do not intend to submit a revision so that we may withdraw it.

**IMPORTANT - SUBMITTING YOUR REVISION**

Your revisions should address the specific points made by each reviewer. Please submit the following files along with your revised manuscript:

1. A 'Response to Reviewers' file - this should detail your responses to the editorial requests, present a point-by-point response to all of the reviewers' comments, and indicate the changes made to the manuscript.

*NOTE: In your point by point response to the reviewers, please provide the full context of each review. Do not selectively quote paragraphs or sentences to reply to. The entire set of reviewer comments should be present in full and each specific point should be responded to individually, point by point.

You should also cite any additional relevant literature that has been published since the original submission and mention any additional citations in your response.

2. In addition to a clean copy of the manuscript, please also upload a 'track-changes' version of your manuscript that specifies the edits made. This should be uploaded as a "Revised Article with Changes Highlighted " file type.

*Resubmission Checklist*

When you are ready to resubmit your revised manuscript, please refer to this resubmission checklist: https://plos.io/Biology_Checklist

To submit a revised version of your manuscript, please go to https://www.editorialmanager.com/pbiology/ and log in as an Author. Click the link labelled 'Submissions Needing Revision' where you will find your submission record.

Please make sure to read the following important policies and guidelines while preparing your revision:

*Published Peer Review*

Please note while forming your response, if your article is accepted, you may have the opportunity to make the peer review history publicly available. The record will include editor decision letters (with reviews) and your responses to reviewer comments. If eligible, we will contact you to opt in or out. Please see here for more details:

https://blogs.plos.org/plos/2019/05/plos-journals-now-open-for-published-peer-review/

*PLOS Data Policy*

Please note that as a condition of publication PLOS' data policy (http://journals.plos.org/plosbiology/s/data-availability) requires that you make available all data used to draw the conclusions arrived at in your manuscript. If you have not already done so, you must include any data used in your manuscript either in appropriate repositories, within the body of the manuscript, or as supporting information (N.B. this includes any numerical values that were used to generate graphs, histograms etc.). For an example see here: http://www.plosbiology.org/article/info%3Adoi%2F10.1371%2Fjournal.pbio.1001908#s5

*Blot and Gel Data Policy*

We require the original, uncropped and minimally adjusted images supporting all blot and gel results reported in an article’s figures or Supporting Information files. We will require these files before a manuscript can be accepted so please prepare them now, if you have not already uploaded them. Please carefully read our guidelines for how to prepare and upload this data: https://journals.plos.org/plosbiology/s/figures#loc-blot-and-gel-reporting-requirements

*Protocols deposition*

To enhance the reproducibility of your results, we recommend that if applicable you deposit your laboratory protocols in protocols.io, where a protocol can be assigned its own identifier (DOI) such that it can be cited independently in the future. Additionally, PLOS ONE offers an option for publishing peer-reviewed Lab Protocol articles, which describe protocols hosted on protocols.io. Read more information on sharing protocols at https://plos.org/protocols?utm_medium=editorial-email&utm_source=authorletters&utm_campaign=protocols

Thank you again for your submission to our journal. We hope that our editorial process has been constructive thus far, and we welcome your feedback at any time. Please don't hesitate to contact us if you have any questions or comments.

Sincerely,

Melissa

Melissa Vazquez Hernandez, Ph.D.

Associate Editor

PLOS Biology

mvazquezhernandez@plos.org

-----------------------------------------

Reviewer #1:

The manuscript by Xue and colleagues explores how phage-targeting antibodies might attenuate the efficacy of phage therapy using an Acinetobacter-targeting phage and immunization of mice with individual phage proteins. Structure-guided antigen screening identified predicted nozzle and tail fiber proteins as responsible for eliciting antibodies that attenuated the efficacy of phage therapy in mice. The manuscript is clearly presented and the experiments are logical and reasonably well described. Nonetheless, the study would benefit from addressing the following comments:

Major comments:

1. The experimental system is somewhat artificial, since patients would never be "pre-vaccinated" with phage before their infection. I understand that this is done to study the effects of antibodies against individual phage proteins, but the translational relevance is still somewhat questionable.

2. The negative staining shown and the resulting aggregate structures proposed is highly spectulative. Conclusions regarding the orientation of phages in aggregates should not be made based only on the TEM data shown in Figure 4. Either the claims need to be softened or additional experiments to show the proposed structures are needed.

3. The assumption is made throughout the manuscript that observed effects from serum of immunized animals is due to the phage- or phage protein-specific antibodies in the serum. These claims would be stronger if antibodies were purified from the serum and tested on their own.

Minor comments:

1. Line 115-116 - Please include a basic description of the mouse model and state the number of mice per group in the text; this information is in the Figure 1 legend but it should appear in the text as well.

2. The fiber and nozzle proteins are sometimes called as such and sometimes called gp40 and gp44, which is confusing and makes it difficult to follow the text at times.

3. The reason for selecting the specific proteins used here for immunization should be described, rather than saying they were chosen based on "previous analysis."

4. Figure 2D-F - These plots appear to have identical values between replicates for many of the samples. This is not expected from ELISA data and raw data are not provided so they can't be assessed.

-----------------------------------------

Reviewer #2:

The question asked by the authors is important, as immune response of animal/human organisms to bacteriophages is still poorly understood. The authors analyzed adaptive immune response to selected proteins of the coat of bacteriophage AbP20, infecting Acinetobacter baumannii. Interestingly, antibodies recognizing some proteins were neutralizing, while those recognizing others did not prevent effective infection of the bacterial host. An interesting hypothesis potentially explaining this diversity is proposed. Nevertheless, major revisions are necessary, and important points must be addressed.

Major critical points:

1. The authors indicated that the AbP20 bacteriophages was characterized in their previous work. However, such a work was not cited. Moreover, I was unable to find any paper describing AbP20 in the literature (using different databases). Therefore, the information is confusing, and it is very unusual to present the whole work based on a subject (bacteriophage in this case) which was not published previously while it is described as being charecterized previously. Therefore, citation of the paper describing characterization of AbP20 is mandatory. If the AbP20 characterization was not published previously, the manuscirpt should be reformatted, and description of the isolation and characterization of this phage must be included.

2. The major problem is interpretation of results presented in Figure 4B. From the presented microphotographs, it is not possible to deduce about the pattern of aggregates, and whether AbP20 virions are connected by ther tails or heads. Cam the authors present micrographs with significantly higher resolution? If not, the conclusions based on pictures shown in Figure 4B are just severe overinterpretation of the results.

Minor critical comments:

3. The title should include names of the bacteriophage and its bacterial host.

4. In experiements with mice, the time course is crucial, and should be mentioned in the Abstract.

5. Line 72 - it would be more appopriate to indicte 80% of patiernts instead of "four patients" or number of patients, instead of percentage, should be indicated in line 70.

6. Line 77 - "a Pseudomonas phage"; it is important to indicate what specific phage are you talking about.

7. Line 80 - the same as in p. 6, but considering "a staphylococcal phage".

8. Line 81 - please indicate what specific protein of T4 is mentioned.

9. Line 89 - Please, be more specific (in fact, this concerns the whole manuscript); what do you mean by "specific immue responses"? In fact, it should be briefly described what pathways of the innate immune response are activated by bacteriophages. Moreover, it should be indicated whether these pathways are complete and lead to massive production of cytokines or thy are somehow silenced.

10. Line 94 - Did you mean "the adaptive immune response" rather then "the immune response"?

11. Line 112 - "AbP20-inducted", instead of "AbP20 induced" (this changes the meaning of the title).

12. Line 165 - Add "genes coding for" after "expressed". Please, remember that genes are expressed, not proteins. Proteins, as products of gene expression, can be produced or synthesized, but not expressed (in other words, only genes are expressed, not proteins; "protein expression" is a jargon which should be avoided).

13. Figure 5 presentes an interesting hypothesis, but the results presented in this paper cannot corroborate it. The presented analysis of micrographs from Figure 4B is an overinterpretation of the results. More convincing micrographs (with significantly higher resolution) should be demonstrated to show testing of this hypothesis.

14. Lines 351-353 - see p. 2 and 13.

15. Description of method should be more precise. Current descriptions lack important details.

16. Line 379 - Where was AbP20 published? See p.1.

17. Line 379 - Replace "logarithmic culture" with "Exponentially growing culture".

18. Line 385 - Provide conditions for ultracentrifugation.

19. Line 405 - Again, genes are expressed, not proteins (see p. 12).

20. Line 406 - Proteins cannot be cloned! The indicated vector is a plasmid, being a DNA molecule, and no protein can be cloned into DNA. Obviously, genes coding for these proteins could be cloned.

21. Supplementary material contains some results showing characterization of AbP20. However, they are just raw results, without any description in the text. This is confusing. Was charactyerization of this phage previously published? If not, building a hypothesis on the basis of weakly characterized model is questionable.

-----------------------------------------

Reviewer #3 (Dwayne Roach):

This is an interesting and potentially impactful study examining how antibodies directed against specific phage structural proteins influence therapeutic efficacy. The single-phage focus is appropriate, and the manuscript provides convincing evidence that antibodies against phage, specifically its gp40 and gp44, are associated with reduced phage activity, increased macrophage uptake, reduced splenic titers, and impaired therapeutic outcomes. The core observation can differentially affect phage therapy outcomes and represents a meaningful contribution to the field.

However, the manuscript does not yet reach the level of mechanistic resolution expected. While multiple phenomena are documented, most are causal relationships between neutralization, aggregation, and immune clearance, and several mechanistic interpretations are therefore stronger than the data directly support. Most conclusions rely on correlated observations across assays rather than direct causal tests. Addressing this, either experimentally and/or through more cautious interpretation with literature support, would substantially strengthen the study.

Major comments

1.Mechanistic relationships between neutralization and clearance are not defined. The central conclusion is that gp40- and gp44-specific antibodies impair therapy through both neutralization and enhanced clearance. While each effect is supported individually, their relative contributions are not established. Whether therapeutic impairment is driven primarily by reduced infectivity, increased clearance, or a combination of both are important determinations, and whether these processes are linked or independent. A clearer dissection is needed.

2.Antibody-dependent aggregation, increased macrophage-associated phage signal, and reduced in vivo titers are all shown. However, it is not demonstrated that aggregation itself drives uptake. The current data do not distinguish between effects of aggregate structure and Fc-mediated opsonization. This distinction is important for the proposed model.

3.In vivo clearance mechanism is inferred rather than directly tested. Reduced splenic phage titers following immunization are consistent with enhanced clearance, but the mechanism is not directly tested in vivo. It is therefore not clear whether phagocytosis, aggregation, or other antibody-dependent processes are responsible.

4.The reduction in EOP is interpreted as inhibition of phage-bacteria interaction, but the affected step is not identified. Neutralization could occur at adsorption, DNA injection, or another stage of infection. Without direct assays, this remains unclear.

5.The manuscript presents multiple related observations (neutralization, aggregation, uptake, and clearance) but these are not fully integrated into a coherent mechanistic framework that would relate to therapeutic settings. The Discussion would benefit from a clearer model and more explicit framing of remaining questions.

6.The dominance of gp40- and gp44-specific effects is likely influenced by the structural organization of this phage. The broader implications across different phages are only briefly mentioned and should be expanded.

Additional comments

7.No endotoxin quantification or toxin controls are provided. Given the reliance on macrophage and immune readouts, this should be clarified or acknowledged as a limitation.

8.The decrease in neutrophil percentage alongside increased total WBC suggests a shift in leukocyte composition rather than normalization. This pattern is not explained, and its restriction to the MOI 10 group is unclear. Absolute counts would improve interpretation. There also appears to be an inconsistency in reported timing (10 h vs 24 h) that should be resolved.

9.The study relies largely on endpoint measurements. Time-resolved data would help establish the sequence of infectivity loss, aggregation, uptake, and clearance.

10.Only total IgG responses are measured. Lack of subclass or Fc-function data limits interpretation of uptake and clearance mechanisms and should be acknowledged.

11.The figures are generally readable, but several multi-panel layouts are visually dense and would benefit from a stronger visual hierarchy or splitting.

# Author comment: Author response to Decision Letter 2

**DOI:** 10.1371/journal.pbio.3004009.r003

25 Jun 2026

Attachment Submitted filename: Response to Reviewers-20260624.docx

# Peer review: Decision Letter 2

**DOI:** 10.1371/journal.pbio.3004009.r004

**Authors:** Melissa Vazquez Hernandez

26 Aug 2026

Dear Dr Yang,

Thank you for your patience while we considered your revised manuscript "Fiber- and nozzle-induced neutralizing antibodies against an Acinetobacter baumannii phage impair phage therapy by inhibiting phage proliferation and promoting immune clearance" for publication as a Research Article at PLOS Biology. This revised version of your manuscript has been evaluated by the PLOS Biology editors, the Academic Editor and the original reviewers.

Based on the reviews, we are likely to accept this manuscript for publication, provided you satisfactorily address the remaining points raised by the reviewers. While R1 suggested an experiment that could strenghten the conclusions, we will not require this, but will require that you adjust your statements accordingly. Please also make sure to address the following data and other policy-related requests.

1) We routinely suggest changes to titles to ensure maximum accessibility for a broad, non-specialist readership, and to ensure they reflect the contents of the paper. Please ensure you change both the manuscript file and the online submission system, as they need to match for final acceptance:

"Antibodies targeting phage fiber and nozzle proteins impair Acinetobacter baumannii phage therapy by blocking infection and promoting immune clearance"

2) Please add the weblink of the funding agencies in the Financial Disclosure statement in the manuscript details.

3) The Ethics statement needs to be a separate, independent (and the first) subheading in the Material & Methods section. It must include the full name of the IACUC/ethics committee that reviewed and approved the animal care and use, as well as the protocol/permit/project license number. https://journals.plos.org/plosbiology/s/ethical-publishing-practice

4) Thank you for providing the numerical data underlying each figure. I might have missed it, but I could not find the data for Fig 2E.

5) Please cite the location of the data clearly in all relevant main and supplementary Figure legends, e.g. “The data underlying this Figure can be found in S1 Data” or “The data underlying this Figure can be found in https://doi.org/10.5281/zenodo.XXXXX”

6) Please ensure that your Data Statement in the submission system accurately describes where your data can be found and is in final format, as it will be published as written there

7) Per journal policy, if you have generated any custom code during the course of this investigation, please make it available without restrictions. Please ensure that the code is sufficiently well documented and reusable, and that your Data Statement in the Editorial Manager submission system accurately describes where your code can be found. More information on our Code Policy, what and how to share can be found here: https://journals.plos.org/plosbiology/s/code-availability

Please note that we cannot accept sole deposition of code in GitHub, as this could be changed after publication. However, you can archive this version of your publicly available GitHub code to Zenodo. Once you do this, it will generate a DOI number, which you will need to provide in the Data Accessibility Statement (you are welcome to also provide the GitHub access information). See the process for doing this here: https://docs.github.com/en/repositories/archiving-a-github-repository/referencing-and-citing-content

As you address these items, please take this last chance to review your reference list to ensure that it is complete and correct. If you have cited papers that have been retracted, please include the rationale for doing so in the manuscript text, or remove these references and replace them with relevant current references. Any changes to the reference list should be mentioned in the cover letter that accompanies your revised manuscript.

In addition to these revisions, you may need to complete some formatting changes, which you will receive in a follow up email. A member of our team will be in touch with a set of requests shortly. If you do not receive a separate email within a few days, please assume that checks have been completed, and no additional changes are required.

We expect to receive your revised manuscript within two weeks.

To submit your revision, please go to https://www.editorialmanager.com/pbiology/ and log in as an Author. Click the link labelled 'Submissions Needing Revision' to find your submission record. Your revised submission must include the following:

- a cover letter that should detail your responses to any editorial requests, if applicable, and whether changes have been made to the reference list

- a Response to Reviewers file that provides a detailed response to the reviewers' comments (if applicable, if not applicable please do not delete your existing 'Response to Reviewers' file.)

- a track-changes file indicating any changes that you have made to the manuscript.

NOTE: If Supporting Information files are included with your article, note that these are not copyedited and will be published as they are submitted. Please ensure that these files are legible and of high quality (at least 300 dpi) in an easily accessible file format. For this reason, please be aware that any references listed in an SI file will not be indexed. For more information, see our Supporting Information guidelines:

https://journals.plos.org/plosbiology/s/supporting-information

*Published Peer Review History*

Please note that you may have the opportunity to make the peer review history publicly available. The record will include editor decision letters (with reviews) and your responses to reviewer comments. If eligible, we will contact you to opt in or out. Please see here for more details:

https://plos.org/published-peer-review-history/

*Press*

Should you, your institution’s press office or the journal office choose to press release your paper, please ensure you have opted out of Early Article Posting on the submission form. We ask that you notify us as soon as possible if you or your institution is planning to press release the article.

*Protocols deposition*

To enhance the reproducibility of your results, we recommend that if applicable you deposit your laboratory protocols in protocols.io, where a protocol can be assigned its own identifier (DOI) such that it can be cited independently in the future. Additionally, PLOS ONE offers an option for publishing peer-reviewed Lab Protocol articles, which describe protocols hosted on protocols.io. Read more information on sharing protocols at https://plos.org/protocols?utm_medium=editorial-email&utm_source=authorletters&utm_campaign=protocols

Please do not hesitate to contact me should you have any questions.

Sincerely,

Melissa

Melissa Vazquez Hernandez, Ph.D.

Senior Editor

mvazquezhernandez@plos.org

PLOS Biology

------------------------------------------------------------------------

REVIEWERS' COMMENTS

------------------------------------------------------------------------

Reviewer #1:

The authors have addressed nearly all of my comments from the initial round of review. Their response to major comment #2 about aggregate structures, however, is not sufficient. Negative stain is highly variable and TEM images of phages often show clustering/aggregation of particles in the absence of IgG. Even with improved images in Fig. 4B, these representative images should not be used to compare aggregate sizes or orientations and attribute differences to phage protein-specific IgG. This issue could be addressed with gold labeling of IgG, or by removing conclusions about phage aggregation and orientation that are only based on the TEM images shown in Fig. 4B.

------------------------------------------------------------------------

Reviewer #2:

The authors addressed all my previous critical comments. I do not have further concerns, and recommend acceptance of the revised manuscript.

------------------------------------------------------------------------

Reviewer #3 (Dwayne Roach):

The authors have responded carefully and constructively to my previous comments, and the revised manuscript is substantially strengthened. In particular, I appreciate the considerable additional experimental work undertaken to distinguish the effects of antibody mediated neutralization from enhanced phage clearance, to examine the stage of the infection cycle affected by antibodies targeting different structural proteins, and to investigate the mechanisms underlying antibody enhanced macrophage uptake. Overall, I consider the major concerns from my previous review to have been satisfactorily addressed.

I have only a few minor suggestions for the final version. First, the authors should use slightly more cautious language when attributing accelerated in vivo phage clearance, since this mechanism is supported indirectly by the in vitro experiments. Second, the data support a role for antibody mediated aggregation in macrophage uptake, but not necessarily a causal role for specific aggregate geometry. Third, the endotoxin level should be described more neutrally, which now reads as a complete lack of potential cofounder effects. These are minor issues and do not affect the main conclusions.

# Author comment: Author response to Decision Letter 3

**DOI:** 10.1371/journal.pbio.3004009.r005

2 Sep 2026

Attachment Submitted filename: Response to reviewers-20260902.docx

# Editor report: Decision Letter 3

**DOI:** 10.1371/journal.pbio.3004009.r006

**Authors:** Melissa Vazquez Hernandez

7 Sep 2026

Dear Hang,

Thank you for the submission of your revised Research Article "Antibodies targeting phage fiber and nozzle proteins impair Acinetobacter baumannii phage therapy by blocking infection and promoting immune clearance" for publication in PLOS Biology. On behalf of my colleagues and the Academic Editor, Michael Laub, I am pleased to say that we can in principle accept your manuscript for publication, provided you address any remaining formatting and reporting issues. These will be detailed in an email you should receive within 2-3 business days from our colleagues in the journal operations team; no action is required from you until then. Please note that we will not be able to formally accept your manuscript and schedule it for publication until you have completed any requested changes.

Please take a minute to log into Editorial Manager at http://www.editorialmanager.com/pbiology/, click the "Update My Information" link at the top of the page, and update your user information to ensure an efficient production process.

PRESS

We frequently collaborate with press offices. If your institution or institutions have a press office, please notify them about your upcoming paper at this point, to enable them to help maximise its impact. If the press office is planning to promote your findings, we would be grateful if they could coordinate with biologypress@plos.org. If you have previously opted in to the early version process, we ask that you notify us immediately of any press plans so that we may opt out on your behalf.

We also ask that you take this opportunity to read our Embargo Policy regarding the discussion, promotion and media coverage of work that is yet to be published by PLOS. As your manuscript is not yet published, it is bound by the conditions of our Embargo Policy. Please be aware that this policy is in place both to ensure that any press coverage of your article is fully substantiated and to provide a direct link between such coverage and the published work. For full details of our Embargo Policy, please visit http://www.plos.org/about/media-inquiries/embargo-policy/.

Thank you again for choosing PLOS Biology for publication and supporting Open Access publishing. We look forward to publishing your study.

Sincerely,

Melissa

Melissa Vazquez Hernandez, Ph.D., Ph.D.

Senior Editor

PLOS Biology

mvazquezhernandez@plos.org
